# GetOrganelle: a fast and versatile toolkit for accurate de novo assembly of organelle genomes

**DOI:** 10.1186/s13059-020-02154-5

**Published:** 2020-09-10

**Authors:** Jian-Jun Jin, Wen-Bin Yu, Jun-Bo Yang, Yu Song, Claude W. dePamphilis, Ting-Shuang Yi, De-Zhu Li

**Affiliations:** 1 Germplasm Bank of Wild Species, Kunming Institute of Botany, Chinese Academy of Sciences, Kunming, Yunnan 650201 China; 2 Center for Integrative Conservation, Xishuangbanna Tropical Botanical Garden, Chinese Academy of Sciences, Mengla, Yunnan 666303 China; 3 Center of Conservation Biology, Core Botanical Gardens, Chinese Academy of Sciences, Mengla, Yunnan 666303 China; 4 Southeast Asia Biodiversity Research Institute, Chinese Academy of Sciences, Yezin, Nay Pyi Taw 05282 Myanmar; 5 Department of Biology, The Pennsylvania State University, University Park, PA 16801 USA

**Keywords:** Assembler, Assembly graph, Plastome, Mitogenome, Organelle genome

## Abstract

GetOrganelle is a state-of-the-art toolkit to accurately assemble organelle genomes from whole genome sequencing data. It recruits organelle-associated reads using a modified “baiting and iterative mapping” approach, conducts de novo assembly, filters and disentangles the assembly graph, and produces all possible configurations of circular organelle genomes. For 50 published plant datasets, we are able to reassemble the circular plastomes from 47 datasets using GetOrganelle. GetOrganelle assemblies are more accurate than published and/or NOVOPlasty-reassembled plastomes as assessed by mapping. We also assemble complete mitochondrial genomes using GetOrganelle. GetOrganelle is freely released under a GPL-3 license (https://github.com/Kinggerm/GetOrganelle).

## Background

The plastid genome (plastome, including the chloroplast and other plastid forms) and mitochondrial genome (mitogenome or chondriome) represent the portions of endosymbiotic organelle inheritance in eukaryotes that have remained in the organelle without being transferred to the nucleus or lost. The plastomes of photosynthetic eukaryotes are generally 120–150 kb in size and typically map as a highly conserved circular and quadripartite structure, with a pair of inverted repeat regions (IRs) that separate the large single copy (LSC) region from the small single copy (SSC) region [1, 2]. Mitogenomes exist in nearly all eukaryotic organisms and vary greatly in genome size and form. To date, six main types of mitogenome organization have been recognized [3]. Animal mitogenomes map as a single circle molecule, ranging from 11 to 28 kb in size and either lacking introns (i.e., type I) or including introns (types II–VI). Fungi and plants have single circular mitogenomes with introns from 19 to 1000 kb in size (type II), or a large and homogenous circular molecule from 20 to 1000 kb in size with small circular plasmid-like molecules (type III), or homogenous linear molecules from 1 to 200 kb in size (type V). Because of their near universal presence and high copy numbers in the cell (individual organelles contain numerous plastome and/or mitogenome copies), organelle DNA markers and genome sequences are easily obtained and have been extensively used for phylogenetic and evolutionary analyses [4–8] and DNA barcoding [9–12]. Since the rapid advances of high-throughput sequencing technologies, sequencing costs have decreased tremendously in recent years. Due to the high copy numbers of organelle genome in a single cell, it is feasible to get enough reads from the low coverage whole genome sequencing (WGS) data to assemble complete organelle genomes [13, 14].

To date, there are ca. 8400 embryophyte plastomes, ca. 65000 animal mitogenomes, ca. 1300 fungi mitogenomes, and ca. 300 plants mitogenomes available in GenBank (accessed on May 15, 2020). Multiple processes or pipelines for assembling organelle genomes have been described, but their assembly qualities vary widely. For example, SPAdes [15], SOAPdenovo2 [16], and CLC Genomics Workbench (https://www.qiagenbioinformatics.com/) have been widely used to assemble the WGS data, after which the organelle genomic scaffolds/contigs were selected or filtered out using a reference genome for further concatenation [17] or post-assembly gap filling and closing [18, 19]. However, these approaches are not only computationally intensive but also error-prone for inexperienced users and complicated samples [20]. The IOGA (Iterative Organellar Genome Assembly) pipeline [21] conducts de novo plastome assembly by incorporating Bowtie2 [22], SOAPdenovo2, SPAdes, and other dependencies for recruiting plastid-associated reads, but the plastomic scaffolds/contigs created in this process need to be finalized by other programs. Fast-Plast (https://github.com/mrmckain/Fast-Plast), NOVOPlasty [23], and ORG.asm (https://git.metabarcoding.org/org-asm/org-asm/org-asm) were proposed as fast tools to conduct de novo assembly of complete organelle genomes from WGS data. However, these tools have not been systematically evaluated until a recent preprint was posted at bioRxiv [24]. Freudenthal et al. [24] presented a benchmark comparison of seven chloroplast assembly pipelines/toolkits and found significant differences among those assemblers. In their tests, our toolkit, GetOrganelle (https://github.com/Kinggerm/GetOrganelle), significantly outperformed all other assemblers in consistency (unlike consistency under different parameters in this paper), accuracy, and success rate. Nevertheless, the broad application and versatile options of GetOrganelle were not explored by Freudenthal et al. [24]. Additionally, organelle genomes may produce flip-flop configurations or other assembly isomers mediated by repeats [25–27]; these outcomes are not addressed by any of the abovementioned pipelines/toolkits, except GetOrganelle. This capability also was not investigated by Freudenthal et al. [24].

**Fig. 1:**
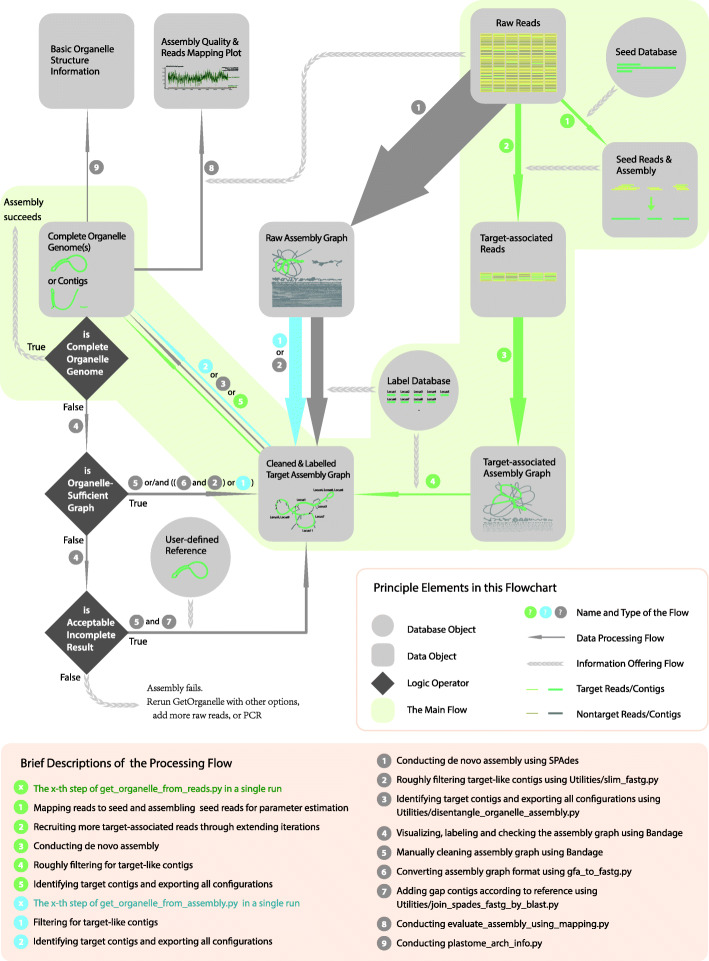
The workflow of the GetOrganelle toolkit. The thumbnails inside data objects show an example of plastome assembly. The solid arrows denote the data processing flows and associated directions, with their width proportional to general computational burden. All green solid arrows together describe a complete run from reads to organelle genome(s), which is encapsulated in a single command using the script “get_organelle_from_reads.py”

**Fig. 2:**
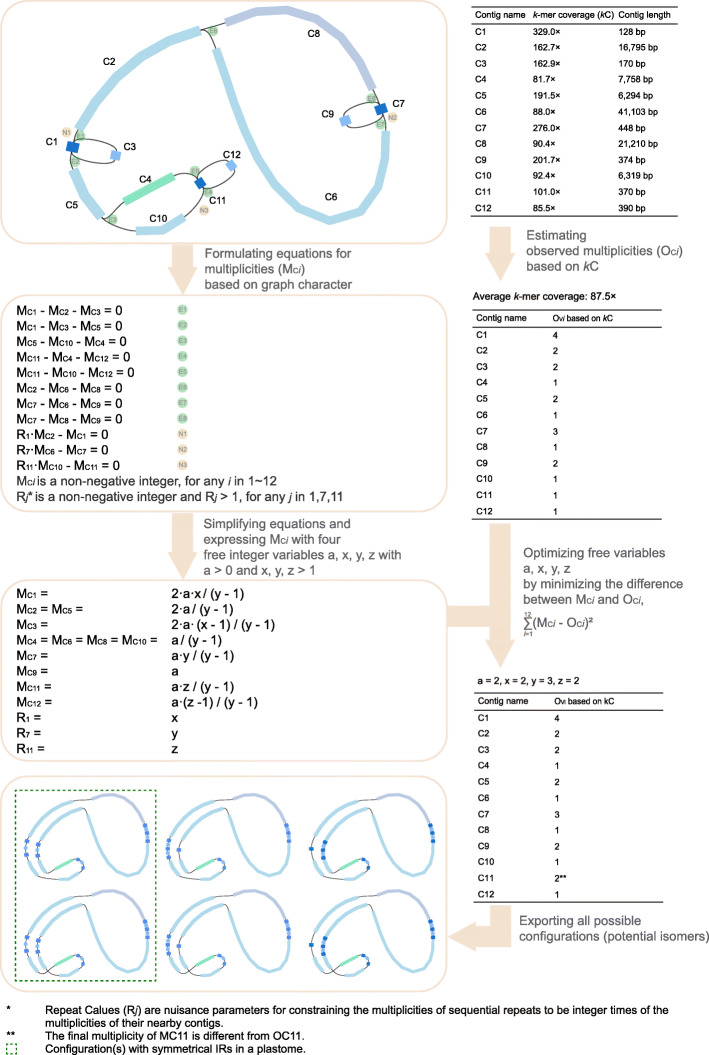
An example of exstimating the multiplicities of contigs and exporting all configurations from a target-complete assembly graph in GetOrganelle (steps 5b and 5c of the script “get_organelle_from_reads.py”)

The GetOrganelle toolkit includes a number of scripts and libraries for recruiting target organelle reads from WGS read data, manipulating and disentangling assembly graphs, and generating reliable organelle genomes, accompanied by labeled assembly graphs for user-friendly manual completion and correction (Fig. 1). The from-reads-to-organelle process can be completed using the script “get_organelle_from_reads.py” with a single line command, which serves as the main workflow of GetOrganelle. This script exploits Bowtie2, BLAST [28], and SPAdes, as well as the Python libraries Numpy, Scipy, and Sympy as dependencies. It starts with recruitment of initial target-associated reads by using Bowtie2 and taking target genome(s) or sequence(s) as the seed; the initial target-associated reads (seed-mapped reads) are treated as “baits” to get more target-associated reads through multiple extension iterations, which is similar in concept to those of the MITObim [29] and IOGA [21] pipelines. However, the core algorithm of GetOrganelle for read extension uses a hashing approach, which cuts the reads into substrings (“Words”) with a uniform length (“Word size”), and adds them to a hash table (“Accepted Words,” AW). During each extension iteration, the AW dynamically increases as new target-associated (overlapped) reads are cut and added as Words. Then, the total target-associated reads are de novo assembled into a FASTA assembly Graph (“FASTG”) file using SPAdes. Non-target contigs in the FASTG assembly are further automatically identified and trimmed by their connections, coverages, and BLAST hit information using a target-gene-based “label” database. The slimmed FASTG file is used to calculate all possible paths of the complete target organelle genome based on the graph characteristics and the coverages of the contigs (see Fig. 2). In some cases, when the assemblies cannot be solved as a circular path or are too complicated to be solved, GetOrganelle will conservatively export the target contigs/scaffolds. Meanwhile, the slimmed assembly graph (FASTG) and selected target assembly graph (GFA) can also be visualized by Bandage [30] to assess the completeness of the final graph, or export selected contigs/scaffolds, or manually remove “noisy” and non-target contig/scaffold connections. The semi-manually cleaned assembly graph can then be used to get the complete target genome or the target contigs/scaffolds using the script “get_organelle_from_assembly.py.” In this study, we have illustrated the mechanism and workflow of GetOrganelle. Moreover, we tested GetOrganelle with a wide range of parameters and samples of plants, animals, and fungi in assembling plastomes and mitogenomes and provide a detailed comparison of the assemblies of NOVOPlasty, the assemblies of GetOrganelle, and the published plastomes.

## Results

### Reassembling plastomes from 50 published plant datasets using GetOrganelle

**Fig. 3:**
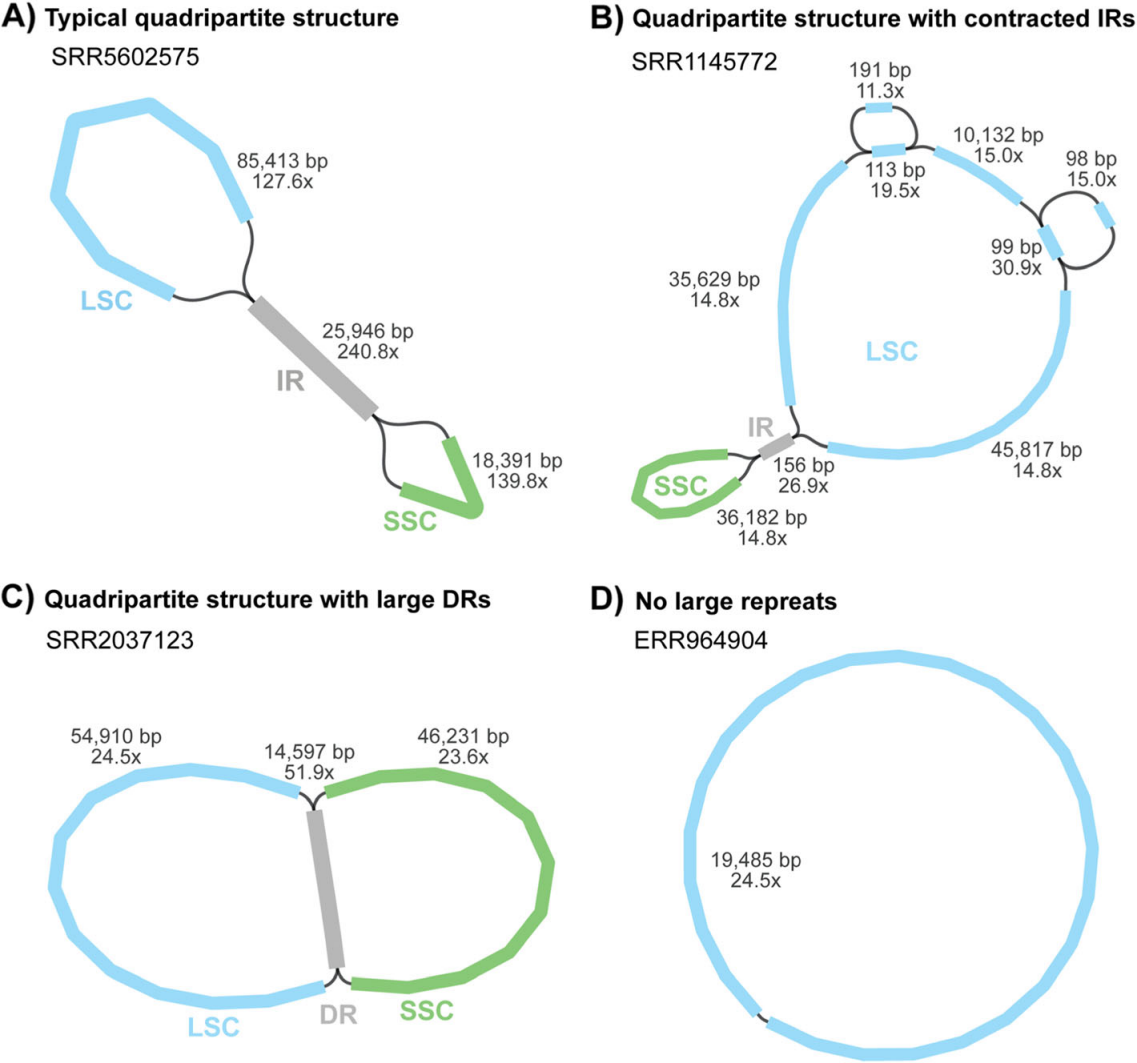
Selected assembly graphs of plastome show quadripartite structures with typical IRs, contracted IRs and large DRs, and no large repeats

**Fig. 4:**
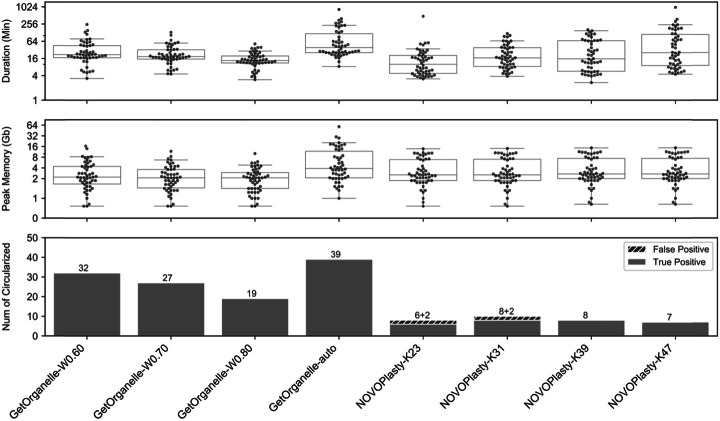
Comparisons of four sets of runs using GetOrganelle and four sets of runs using NOVOPlasty when assembling 50 public plant datasets. The labels along the bottom denote the program and key settings for each set of 50 runs. The dots in the upper boxplot show the time cost in seconds for finishing each run. The dots in the middle boxplot show the maximum memory usage in gigabytes for finishing each run. The bottom histogram shows the number of circularized genomes for each set

GetOrganelle reassembled the complete circular plastome(s) from 47 out of the 50 plant datasets (Additional file 2: Table S1), including samples with typical IRs, contracted IRs, no IRs, and large direct repeats (DRs) (Fig. 3). Of the remaining three species, two species (*Ginkgo biloba* L., ERR2206741 and *Salvinia cucullata* Roxb., SRR6478596) had two break points in the LSC/SSC region, and one species (*Musa balbisiana* Colla, SRR2057084) consisted of 14 plastome fragments (Additional file 2: Table S1; see details at https://github.com/Kinggerm/GetOrganelleComparison version 1.1.1). The GetOrganelle-reassembled complete circular plastomes were identical to the published ones in 14 samples, and different from published plastomes by fewer than five site-differences and/or less than 100-bp differences in 30 other samples (Additional file 2: Table S1, Table S2). Most of the differences were due to repeats or indels. Read mapping generally supported the GetOrganelle-reassembled plastomes more than the published plastomes (Fig. 4; Additional file 2: Table S2). For example, the IR boundary regions of *Laurus nobilis* L. (SRA: SRR5602602; GBK: KY085912.1), which showed significant assembly differences between the published and GetOrganelle-reassembled plastomes, had a smooth mapping plot for the GetOrganelle plastome but a drastically fluctuating mapping plot for the published one (Additional file 1: Fig. S1). Those differences can also be monitored from the summarized mapping quality (Additional file 2: Table S2), where the GetOrganelle-reassembled plastome had similar matched depth to the published plastome (221.60 vs 221.65), with a much smaller standard deviation (31.43 vs 37.88), and a smaller error rate (1.16% vs 1.17%) with a much smaller deviation (1.11% vs 1.56%) (see the “Methods” section for the calculation).

**Fig. 5:**
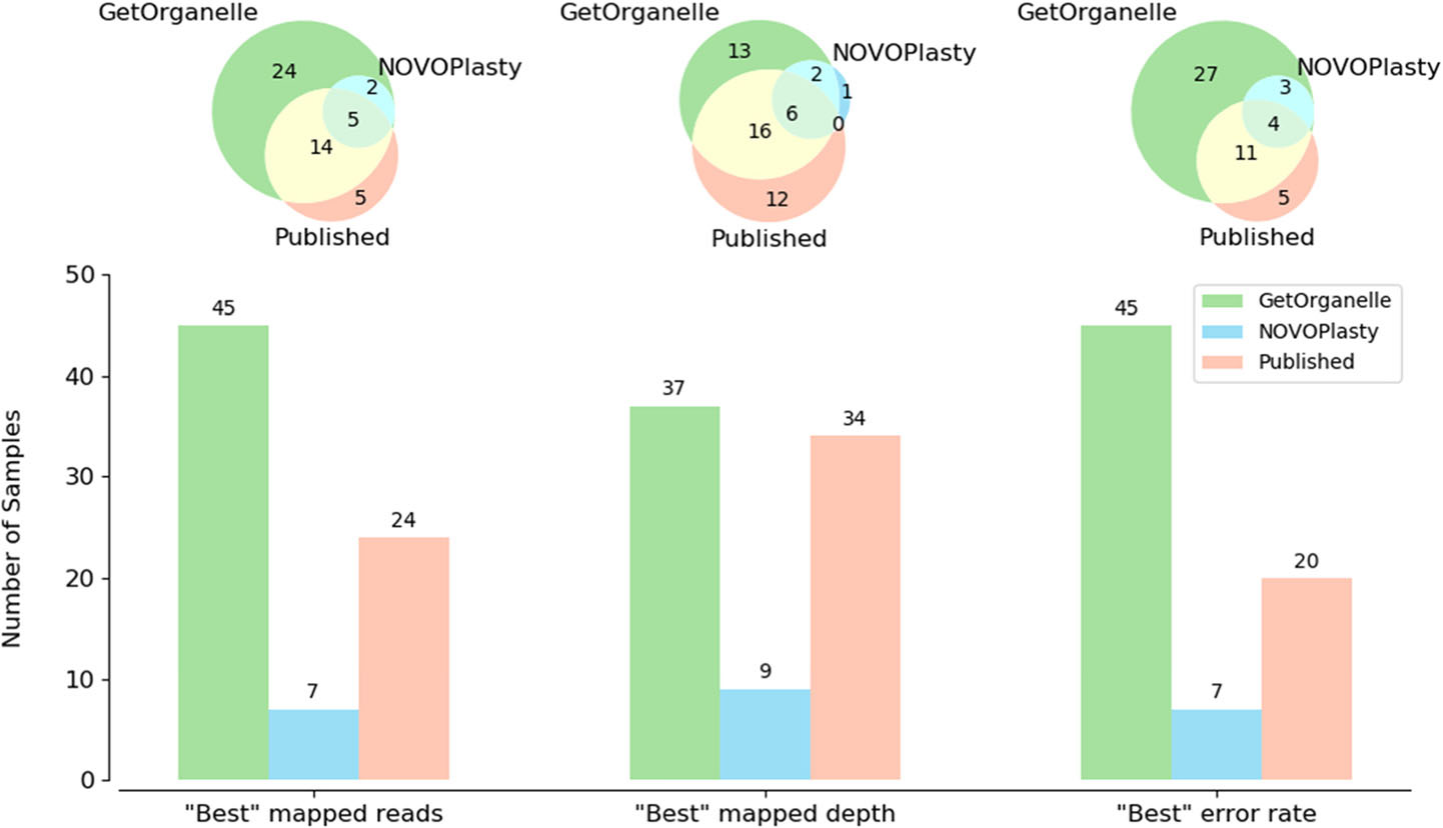
Evaluating assembly qualities of GetOrganelle plastomes, NOVOPlasty plastomes, and published plastomes using read mapping

Evaluations of assembling the 50 plant datasets showed that the mean memory, the mean duration, and the number of circularized genomes all decreased with the word size ratio (WSR, defined as the Word size over the effective mean read length) adjusted from 0.6 to 0.8 (Fig. 5). Runs in an “auto” mode using an automatically estimated word size for read extension generated circular plastomes in 39 samples ranked as the highest success ratio among the tested parameter sets, though “auto” mode also had the most intense computational usage in both mean memory and duration (Fig. 5). Eight more datasets were assembled into complete circular plastome(s) by adjusting the Word size for extension, and/or adjusting the *k-*mer values for de novo assembly using SPAdes, and/or using other empirical options (see details at https://github.com/Kinggerm/GetOrganelleComparison version 1.1.1).

GetOrganelle exported all possible configurations mediated by potential flip-flop recombination induced by IRs [31] or other shorter repeats. When a complete circular plastome was obtained, GetOrganelle generally outputted consistent assemblies with the same raw reads using different parameters. In the 50 plant datasets, there were 33 samples that resulted in complete circular plastomes reassembled from multiple runs using different parameters of GetOrganelle. Twenty-five out of 33 samples produced identical assemblies using different parameters. Five out of 33 samples (SRA: SRR5602586, SRR5602590, SRR5602594, SRR5602605, SRR6932851; we will use the SRA number to represent the relevant samples thereafter) had assemblies that diverged where there were 1- or 2-bp indels. For SRR5028199, the plastome of the GetOrganelle-W0.6 run is longer than the plastome of any other three runs in a 19-bp repeat (“GAAAAGAAAGAATGAGAAA”). For SRR5602597, the plastome of the GetOrganelle-W0.8 run is shorter than the plastome of any other three runs in a 36-bp repeat and a 273-bp indel/repeats. For SRR5602577, the plastome of the GetOrganelle-W0.8 run is shorter than the customized run in a 6-bp poly-A/T indel.

### Reassembling plastomes from 50 published datasets using NOVOPlasty

For reassembling plastomes from the same 50 plant datasets (see Additional file 2: Table S1) using NOVOPlasty, only 15 samples were “claimed circular” (i.e., eight for *k-*mer 23, ten for *k-*mer 31, eight for *k-*mer 39, and seven for *k-*mer 47) (Fig. 4; Additional file 2: Table S2). Of the “claimed circular” samples, three samples had gaps or ambiguous sites in K23 runs, six samples had gaps or ambiguous sites in K31 runs, five samples had gaps or ambiguous sites in K39 runs, and five samples had gaps or ambiguous sites in K47 runs (see details at https://github.com/Kinggerm/GetOrganelleComparison version 1.1.1). In addition, some claimed circular samples were not really circularized. For example, there are two contigs in both the K31 run of SRR5602581 and the K47 run of SRR5602590. Some claimed circularized runs (such as the K23/K31 runs of SRR2037123, and the K23 run of SRR5602581) were problematic because the lengths of assemblies deviated strongly from both the published plastomes and the reassembled plastomes using GetOrganelle. In the case of SRR2037123, NOVOPlasty lost the entire large single copy region between a pair of direct repeats and produced a plastome that was “reduced” in size by 60,647 bp (46.7%). Of the remaining 12 samples for which NOVOPlasty obtained reasonable lengths, the read mapping qualities for NOVOPlasty assemblies were good though generally not better than those of GetOrganelle, especially in the error rate (Fig. 4; Additional file 2: Table S2). Only three datasets (SRA: ERR1917165, ERR964904, and SRR5602589) were assembled by NOVOPlasty into plastomes that were identical to the published ones (Additional file 2: Table S2).

The average duration of NOVOPlasty runs increased as the *k-*mer values increased from 23, through 31 and 39, to 47 (marked as K23, K31, K39, K47 runs separately), while the average memory cost was very close among the four *k-*mer values (Fig. 5). The average duration of NOVOPlasty K23 and K31 runs was generally shorter than that of GetOrganelle runs. The average duration of K39 runs was similar to that of GetOrganelle-W0.8 runs. The average duration of K47 runs was longer than that of GetOrganelle runs except for GetOrganelle-auto runs. The average memory cost of the NOVOPlasty runs was lower than those of GetOrganelle-W0.60 and GetOrganelle-auto runs, but greater than those of GetOrganelle-W0.7 and GetOrganelle-W0.8.

Additionally, the assemblies of NOVOPlasty are largely inconsistent and varied greatly when using different *k-*mer values. In nine samples, NOVOPlasty obtained the complete circular plastome in multiple runs. However, NOVOPlasty generated consistent assemblies using four *k*-mer values in only three samples (SRA: ERR1917165, SRR5602589, and SRR5602602) out of the nine.

### Assembling mitogenomes using GetOrganelle and NOVOPlasty

For 56 animal mitogenome datasets, GetOrganelle and NOVOPlasty successfully assembled 29 and 23 complete circularized mitogenomes, respectively, with the overlap of 19 datasets (Additional file 2: Table S3). Of the 19 datasets, GetOrganelle and NOVOPlasty generated mitogenomes of similar sizes (≤ 2 bp differences) in 14 samples. GetOrganelle failed to call any animal mitogenome contigs in four samples, while NOVOPlasty used the same seed but failed in nine samples.

The average number of genes (number of genes here refers to number of gene hits based on BLAST search; the same below) is 10.61 for GetOrganelle animal mitogenome assemblies, and 8.38 for NOVOPlasty assemblies. In 10 samples that GetOrganelle succeeded and NOVOPlasty failed, NOVOPlasty assemblies called 70 genes in total while GetOrganelle called 114 genes. In those four samples that NOVOPlasty succeeded but GetOrganelle failed, GetOrganelle assemblies called 31 genes in total while NOVOPlasty assemblies called 45 genes.

For 50 fungal mitogenome datasets, GetOrganelle and NOVOPlasty successfully assembled 24 and 26 complete circularized mitogenomes, respectively, with 21 being assembled by both programs (Additional file 2: Table S4). Concerning the generated complete mitogenome size, GetOrganelle and NOVOPlasty generated closely similar sizes (< 15-bp difference) from 19 of the samples. In SRR5804015 that both GetOrganelle and NOVOPlasty claimed circularized results, NOVOPlasty lost a contig of 9606 bp in between a pair of direct repeats and lost seven functional genes such as *nad1* and *apt6*.

The average number of genes is 17.32 for GetOrganelle fungal mitogenome assemblies, and 12.76 for NOVOPlasty assemblies. In three fungal samples (SRR5803930, SRR5804127, and SRR5804147) that GetOrganelle succeeded and NOVOPlasty failed, NOVOPlasty generated highly fragmented contigs with unreasonable total lengths and only 15 genes in total, comparing to 48 genes from three GetOrganelle assemblies. On the contrary, in those five samples that NOVOPlasty succeeded but GetOrganelle failed, GetOrganelle generated a nearly complete or comparable result with NOVOPlasty, losing only one gene out of the 94 genes detected in five NOVOPlasty assemblies.

### Features of GetOrganelle

**Fig. 6:**
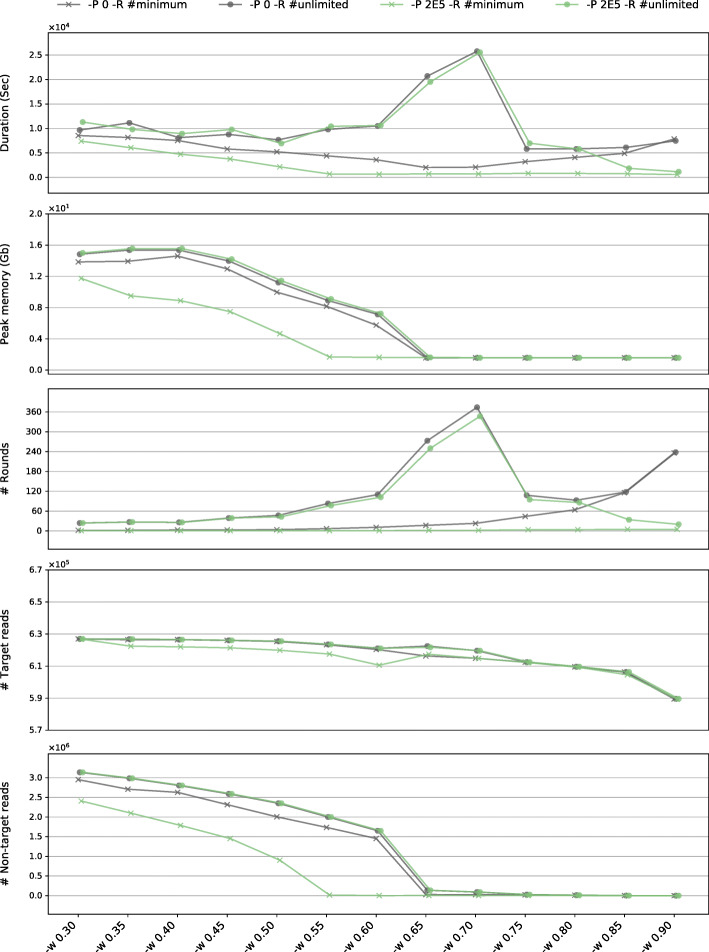
Assessing the plastome assembly performance characteristics of GetOrganelle using online reads of an angiosperm species, *Haberlea rhodopensis* (SRA: SRR4428742) as the dataset, using the complete plastome of a gymnosperm species, *Gnetum parvifolium* (GBK: NC_011942.1) as the seed. The assessment was conducted across a range of Word sizes (in the form of WSR, after the flag “-w” along the *x* axis). The green marks denote using the pre-grouping value 200,000 (after the flag “-P”), while the gray marks denote that the pre-grouping was disabled. The cross marks denote using the minimum number of rounds of extension iterations for achieving a complete plastome or stabilizing the incomplete plastome result, while the solid circle marks denote using unlimited number of rounds (“-R 1000” in practice). From top to bottom, for assembling raw reads into the same complete plastome (or the same incomplete result in all runs of “-w 0.90”), the five subgraphs present the total computational time cost in seconds, the maximum memory cost in gigabytes, the actual number of rounds GetOrganelle took, the number of target plastid reads GetOrganelle recruited, and the number of non-target reads GetOrganelle recruited, respectively

To explore the influences of parameters and seed, we assembled reads of an angiosperm species, *Haberlea rhodopensis* Friz. [5M 100-bp paired-end Illumina reads; GenBank Sequence Reads Archive accession number (SRA): SRR4428742], using the complete plastome or a short plastomic fragment *rbcL* gene of a gymnosperm species, *Gnetum parvifolium* (Warb.) W.C. Cheng [GenBank accession (GBK): NC_011942.1] as the seed (Fig. 6, Additional file 1: Fig. S2, Additional file 2: Table S5). The runs for all tested WSR, except for 0.9, assembled an identical complete plastome, no matter whether the initial seed was a complete plastome or a short plastomic fragment *rbcL* gene, and regardless of whether pre-grouping (an ad hoc speeding-up algorithm, see more in the “Methods” section) was enabled or not.

The run duration of GetOrganelle varied as the WSR set from 0.30 to 0.90 (Fig. 6). The duration of the runs with unlimited number of rounds (lines with circle marks in Fig. 2) increased drastically when the WSRs were 0.65 and 0.70. The duration of the runs with minimum number of rounds and pre-grouping disabled (gray line with cross marks) decreased when the WSR ranged from 0.30 to 0.65, while it increased with WSR ranging from 0.70 to 0.90. The time cost of the runs with the minimum number of rounds and pre-grouping enabled (green line with cross marks) decreased when the WSR ranged from 0.30 to 0.55, but it did not increase when using large WSRs. The maximum memory occupation generally decreased when the WSR was smaller than 0.65 and remained unchanged when the WSR reached 0.65. A too large WSR, such as 0.90 in our test, risked producing an incomplete result.

When testing with unlimited number of rounds, the runs with pre-grouping enabled (gray line with circle marks) and the runs with pre-grouping disabled (gray line with cross marks) recruited exactly the same number of the final accepted reads (Fig. 6); thus, they generated the same final assemblies and similar computational durations and memory costs. However, when considering the runs with minimum rounds, the pre-grouping runs generally needed significantly fewer rounds of extension (< 6) to recruit most of the target-associated reads to achieve a complete plastome or to stabilize an incomplete result. Extension beyond the first five rounds is mostly to recruit non-target reads. By cutting off the latter unnecessary rounds of extension with minimum rounds (lines with cross marks), the pre-grouping runs (green line with cross marks) can reduce both time and memory cost, sometimes significantly.

## Discussion

### Accuracy of the published/reassembled plastomes

GetOrganelle-reassembled plastomes contained identical sequences to the published plastomes in 14 of 50 plant samples, including a lycopod species, *Selaginella kraussiana* (Kunze) A. Braun (SRR2037123) that was reported to have large DRs [32]. Read mapping plots of GetOrganelle-reassembled complete or near complete plastomes are generally smooth. In contrast, the NOVOPlasty-reassembled plastomes (e.g., ERR964904, SRR2037123, SRR5602581, as mentioned above) had unreasonably shorter lengths, but were claimed to be circularized. In addition, read mapping plots (see https://github.com/Kinggerm/GetOrganelleComparison/tree/master/eval/Published version 1.1.1) showed that some published plastomes (e.g., SRR1145775, SRR2057084, SRR5602601, SRR5602602, SRR6478596, SRR7630500) have incorrect IR boundaries or contig overlaps as revealed by the local coverages dramatically increasing or decreasing in the regions or sites.

Our evaluation using read mapping (Additional file 2: Table S2) showed that 37 of 50 assemblies generated by GetOrganelle had the best-ranked matched depths (smaller average and smaller deviation). For three samples (SRR2057084, SRR6478596, ERR2206741), GetOrganelle failed to generate a circular plastome. For SRR2037123, NOVOPlasty had the best-ranked matched depths but produced a problematic assembly. Of the remaining ten samples that GetOrganelle did not have the best-ranked matched depths, all the GetOrganelle-reassembled plastomes have the best-ranked error rate (smaller average and smaller deviation) and the best-ranked or nearly-best-ranked number of mapped reads.

Our evaluation using read mapping (Additional file 2: Table S2) showed that 45 assemblies using GetOrganelle had the best-ranked error rate; the remaining five assemblies, of which three assemblies were not circular, had values very close to the best-ranked ones. However, many published plastomes (e.g., SRR5602575-SRR5602578, SRR5602581-SRR5602584, SRR5602587, SRR5602588, SRR5602592, SRR5602593, SRR5602595, SRR5602597, SRR5602598, SRR5602599, SRR5602600, SRR5602609, SRR5602610, SRR6932851, SRR7630500) have nucleotide sites that are incongruent with the consensus of most mapping reads, as revealed by extremely high mismatch/indel rates at some sites, identifiable as extremely outlier points in the mapping plot.

Our results also showed that GetOrganelle had outstanding consistency, indicating that exact parameter settings are not required to obtain an accurate assembly. Of the 33 samples reassembled to complete circular plastome by multiple runs, there are 30 samples that had identical or nearly identical (≤ 2 bp difference) results using different parameter values. There are three main reasons for the discrepancies in the remaining few samples. First, a large Word size recruits significantly fewer reads (e.g., -w 0.8 in SRR5602597) or may even cause an incomplete result (e.g., -w 0.8 in SRR5028199, SRR5602590, and SRR5602594, and -w 0.9 in the performance test). Second, there were small repeats that could not be precisely identified by low local coverage, which might be the reason for inconsistency in SRR5028199. Third, there were polymorphisms in the data causing an incomplete result, such as in SRR5602577 recovered by the auto mode.

Our results also suggest that using read mapping quality is an easy and important approach of evaluating organelle genome assemblies. We tested 50 plant datasets here, not only to show that GetOrganelle has higher accuracy for assemblies than other currently available assembly toolkits, but also to argue for the necessity for all plastome providers to make raw data available to the public (e.g., SRA); this will allow assemblies to be reproduced, evaluated, and amended to benefit future comparative analyses of organelle genomes [33].

### Large inverted, direct repeats and “weird” plastome assembly

The entanglement of repeats is one of the challenges in assembling plastomes, though none of the reported plastome assemblers has addressed this challenge. The largest repeat in a canonical plastome is a pair of generally identical inverted sequences that usually contain the ribosomal DNA genes, and is known simply as the inverted repeat (IR). Given that flip-flop recombination mediated by the IRs is common [31, 34], a genuine de novo assembler such as GetOrganelle should export at least two configurations when IRs exist. Even if we assume that there was only one configuration in vivo, short sequencing reads theoretically cannot tell the difference between those two configurations when IRs exist. However, for example, NOVOPlasty produces, when successful, only one representative of the plastome structure, which risks misleading less experienced researchers to treat SSC orientations as an important inversion [34].

A key issue concerning IR recovery in plastid genome assembly is the identification of the IR boundaries. A traditional assembly method, which uses CLC or another assembler to make sequence contigs and complete the circular assembly based on a reference, is prone to create a plastome with a similar IR length/boundary as the reference. In that case, PCR verification was required for the four boundaries of a canonical quadripartite plastome in conventional assemblies. The concept of the necessity of PCR verification still overshadows many empirical studies in the NGS age. However, our graph-based method for completing the circular plastome uses the original contig connections that are supported by actual read overlaps. When sufficient read coverages (e.g., 100×) support the contig connections between the boundaries, there would be neither need for PCR verification or read mapping for IR boundary identification. Based on the nature of the overlap of short reads, the credibility of the IR boundaries is actually the same as that of other parts of the plastome. For a genuine organelle genome de novo assembler, the IR boundaries can be constructed purely from read information rather than any references, the same as any other region of the genome. As evidence, GetOrganelle correctly recovered the extreme contraction or loss of a large pair of IRs in *Juniperus cedrus* Webb & Berthel. (SRR1145775) and two *Picea* species (ERR268390, SRR5028199). Another interesting example is that GetOrganelle successfully assembled the plastome of *Selaginella kraussiana* (SRR2037123) with two large DRs, without being supplied prior knowledge of the IRs or DRs. However, for the same sample, NOVOPlasty only achieved part of the complete plastome, lost one copy of the large DRs and one large single copy region, and claimed it to be circular (another similar inaccurate NOVOPlasty result was the mitogenome assembly of SRR5804015). Our reassembled plastome is identical to the published one (MH549643.1), which was assembled using Velvet and SPAdes, and then verified using PCR [32]. For an IR lacking species, such as a species in the IR-lacking clade of Fabaceae, GetOrganelle could correctly generate a plastome without IRs, with a single circle assembly graph as visualized by Bandage (e.g., R. Zhang et al., unpublished data).

It is a challenge to assemble some “weird” plastomes (e.g., reduced plastomes, plastomes with gene translocations, IR expansions, contractions, or losses) using some traditional methodologies [20]. For example, non-photosynthetic plants typically have reduced plastomes, pseudogenes, inversions, and gene translocations [20, 35, 36]. We successfully assembled a complete and yet highly reduced plastome without IRs for the holoparasite *Cytinus hypocistis* (L.) L. (ERR964904) using a customized strategy, excluding contigs with an obvious false hit. We also were able to assemble circular plastomes for many holoparasites from Balanophoraceae, Convolvulaceae (e.g., *Cuscuta* spp.), Lennoaceae, and Orobanchaceae, as well as hundreds of hemiparasites using GetOrganelle (W.-B. Yu et al., unpublished data). Another type of “weird” plastomes are those carrying insertions of mitochondrial DNA, such as carrot [37], milkweed [38], and bamboo plastomes [39]. For such plastomes, we also assembled accurate plastomes with the mitochondrial insertions (additional tests via https://github.com/Kinggerm/GetOrganelleComparison version 1.1.1; based on 20M reads of SRR2147183 and 30M reads of SRR4243000) using GetOrganelle without any prior hints of that insertion. In the “embplant_pt” mode, GetOrganelle keeps both chloroplast contigs and mitochondrial contigs in the post-slimmed assembly graph, so that chloroplast and mitochondrial contigs can be more accurately and reasonably distinguished based on not only blast hit, but also contig coverage and contig connections. If there is strong coverage or graph character evidence for a mitochondrial insertion to be an indispensable part of the plastome, GetOrganelle will keep that insertion.

### Mitogenome assembly of fungi and animals

Despite similar mitogenome circularizing ratio, using the same seed, GetOrganelle was generally better over NOVOPlasty in obtaining mitogenome contigs and genes, even in samples with a relatively low mitogenome coverage (Additional file 2: Table S3, S4). Besides, in the fungal mitogenome assembly of SRR5804015, NOVOPlasty lost a contig of 9606 bp with seven functional genes in between a pair of direct repeats, in addition to the large-scale loss in the *Selaginella kraussiana* (SRR2037123) plastome assembly. The early termination of sequence extension might be an issue underlying the algorithm of NOVOPlasty.

The mitogenomes of fungi and animals usually have a much higher nucleotide substitution rate than plastomes [40, 41]. Therefore, unlike assembling plastomes, a relatively closely related seed or label database would be indispensable for assembling and identifying mitogenomes using the extension and assembly strategy of GetOrganelle. If GetOrganelle fails using the default database for animals or fungi mitogenome assembly, we suggested that users rerun the same sample using their own seed and label databases.

Although GetOrganelle has demonstrated success at separating different organelle components (e.g., separating plastome and mitogenome components in complex assembly graphs in samples such as SRR5602593 and ERR1917165) and repeat resolution, this ability of GetOrganelle largely relies on an accurate assembly graph output by SPAdes. The only run in these tests that violated this prerequisite was the fungal mitogenome assembly of SRR5802125, which generated abnormal connections that in turn distorted the multiplicity estimations of contigs. Although the assemblies using both tools obtained the same gene hits, the contig multiplicities estimated by GetOrganelle are obviously disproportionate to the contig coverages. This issue could be solved by incorporating different assembly engines and reporting the disproportionality of contig multiplicities over the contig coverages to warn users, a feature planned for a future version of GetOrganelle.

### Other small repeats, broken graph, and manual completion

When attempting to solve the organelle genome structure, other small repeats can cause awkward tangles in the assembly graph. Plastomes of some plant clades have been reported to have multiple short repeats, such as some clades in Ericaceae [42, 43], Geraniaceae [44], and Pinaceae [26, 45]. Most plant mitogenomes contain a high number of short repeats [46–48]. In contrast, mitogenomes in animals and most fungi are contracted and generally lack repeats [49]. However, in our trial analyses, we did discover a few samples of fungi (e.g., SRR5801935, *Macrolepiota dolichaula* (Berk. & Broome) Pegler & R.W. Rayner, SRR5802125, *Collybia* sp., and SRR5804018 *Grifola frondosa* (Dicks.) Gray) and animals (e.g., SRR136494, *Mayetiola destructor* Say, SRR4340274, *Daphnia magna* Straus, SRR1298377, *Halyomorpha halys* Stål) that have multiple repeats, which may result in isomeric assemblies. Organelle genomes with many short or long sequence repeats, such as the plastomes of Chlamydomonas and some Ericaceae and Geraniaceae species, may be intractable for short-read sequencing data. With such genomes, GetOrganelle may generate tens of thousands of circular sequences or highly fragmented contigs; long read data such as PacBio HiFi data may provide a solution for improved automated assembly in these difficult cases.

One common feature of organelle genomes is tandem repeats, which can be visualized as whirls in the assembly graph. The main challenge with disentangling tandem repeats in an assembly graph is determining the multiplicity (copy number) of the contigs. Many bacterial genome assemblers use the minimum or the greedy algorithm for detecting multiplicity, which is simply assuming every tandem repeat contig has multiplicity two and constructing the assembly as a path that traverses the repeat twice [50–52]. GetOrganelle disentangles the tandem repeats based on both the connection information and the contig coverage, a more reality-based approach, which has a better chance of success if the target organelle genome has some tandem repeats of more than two copies and the reads are of sufficiently high coverage to overcome the error (Fig. 2). Other repeats, such as short inverted repeats other than the large IRs, cause not only assembly graph tangles, but could also mediate recombination, potentially producing isomers in the same individual [25–27, 53]. For researchers studying plastome rearrangement and recombination dynamics, GetOrganelle is the right tool to exhaust the limits of the assembly graph and presents all possible configurations, such as plastomes of *Juniperus cedrus* (SRA: SRR1145775) and *Picea* species (ERR268390, SRR5028199) and mitogenomes of some fungal (SRR5801935, SRR5804018) and animal species (SRR4340274, SRR136494). Currently, the potential configurations mediated by short repeats need to be confirmed by read pair mapping or PCR verification. With the development of long insert size libraries and long-read sequencing platforms (e.g., PacBio), these data can be added for improving repeat resolution and configuration confirmation. A function that incorporates long library reads or long-read sequencing data in estimating the proportion of all candidate isomers is planned for future versions of GetOrganelle.

Some assembly graphs may not be organelle-sufficient (broken graph) or are too complicated to estimate multiplicity. In such cases, users are recommended to perform manual completion or manual multiplicity estimation with the simplified assembly graph using Bandage, along with the target-hit-contig table from the concomitant, cognominal TAB-formatted file. An advantage of manual completion is that it is possible to detect sequence interchange between the nuclear genome, mitogenome, and plastome. This is especially the case when multiple organelle genome mode is selected for plant datasets. The result could be a tangly assembly graph mixed with plastome, mitogenome, and plastome-mitogenome-shared contigs. If de novo assembly and manual completion cannot yield a complete sequence of the plastome, gap filling and PCR verification are needed [20, 42].

### Seed and label database in GetOrganelle

GetOrganelle does not rely on having a closely related plastome for successful assembly; in fact, it is possible to accurately assemble the plastome from short reads of an angiosperm species, *Haberlea rhodopensis*, with high and homogeneously distributed plastid coverage, using the plastome of a gymnosperm species, *Gnetum parvifolium*, as the initial seed. For the same set of reads, using a short, conserved DNA fragment (*rbcL*) as the seed is sufficient to recruit almost all target-associated reads to assemble a complete plastome, but less efficient. The reason for this is that the seed is only used for read mapping to collect the initial target-associated reads, after which the seed will no longer be used (Fig. 1). GetOrganelle then uses the initial target-associated reads to recruit more target-associated reads using multiple rounds of extension, which is mainly based on the nature of the read overlaps with the previously recruited target-associated reads, rather than using the seed as a reference in assembly process. Nevertheless, using the organelle genome of a non-related species as the seed can only be applied to plastome seed recruitment, but not to animal and fungal mitogenome seed recruitment due to their high divergences owing to rapid rates of sequence evolution (see the section “Mitogenome assembly of fungi and animals”).

Since the label database (see Fig. 1) in GetOrganelle is used to provide a supervision signal for labeling contigs prior to semi-supervised learning, the label database should come from conserved regions, such as coding regions, but not necessarily a complete set of genes. After labeling, GetOrganelle uses an integrated strategy (see the “Methods” section) to identify target contigs, which largely relies on the contig coverages and assembly graph characteristics. Thus, the label database of gene fragments has limited influence on the final organelle-complete graph output and would have only a minor effect on the scaffolding of organelle contigs, except for cases where the assemblies have no hits against the database.

### Word size in GetOrganelle

Word size, like the *k-*mer in De Bruijn graph-based assembly, is important to the feasibility and efficiency of read extension, but it differs from *k-*mer in providing the overlap threshold for data filtering. The smaller the Word size is, the lower the threshold is. The use of Word size not only does examine the nature of connection between any two target reads, but also in a way assesses the connection strength by the coverage. Even if the plastome shares similar sequence with mitogenome or nuclear genome, the reads of those non-target genomes would not be largely recruited due to the low coverage, meaning that any two reads of those non-target reads are less likely to be connected in the shallow coverage WGS data.

The Word size is generally negatively correlated with memory usage (Fig. 6), while *k*-mer is generally positively correlated with memory usage. Memory cost decreases when the Word size increases because the larger the overlap threshold (causing fewer reads to be connected and recruited based on the overlap), the smaller the word space during extension. Thus, *k-*mer only influences the assembly process, while the Word size influences the extension process, the numbers of reads to be recruited, and the assembly process. For many real datasets, because the plastome might share homologous regions with non-target genomes [37–39], a small Word size means that a relaxed threshold would not promptly stop the extension when the extension was walking through the non-target genomes. In the case shown in Fig. 6, using a WSR < 0.65 wastes computational resources.

The duration of a whole assembly run is affected by the Word size in various ways (Fig. 6). Generally, with unlimited number of rounds, there are two significant but antagonistic effects shaping the curve of the duration against the Word size (or in the form of WSR). The first one is the “slowing-speed effect”, meaning that a larger Word size means smaller steps in extension and consumes more rounds and more time to recruit the same set of reads. The second one is the “reducing-data effect,” meaning that a larger Word size also results in a briefer extension period with fewer non-target reads, reducing the run time in de novo assembly and other downstream calculation. In the performance test (Fig. 6), a typical manifestation of both effects was where the time cost remarkably increased at WSRs in 0.65 and 0.70, which was caused by the relatively slow speed in extension and a relatively high amount of non-target reads to recruit. In this test, using the minimum number of rounds could cut off most non-target reads and eliminate the “reducing-data effect” in a slow-paced extension (a relatively large WSR, i.e., ≥ 0.65 in the pre-grouping disabled case and ≥ 0.55 in the pre-grouping enabled case), but cut off less non-target reads as the WSR become smaller (Fig. 6). Therefore, when pre-grouping was disabled and the minimum rounds were run, the plot of duration against WSR was not a monotonic rising curve (“slowing-speed effect”) but suffered the “reducing-data effect” when the WSR < 0.65 (Fig. 6). Using the pre-grouping algorithm could generally eliminate the “slowing-speed effect”. Consequently, in this test, the time cost is nearly constant when the WSR ≥ 0.55, but still suffers the “reducing-data effect” when the WSR < 0.55 (Fig. 6). In empirical studies, the minimum number of rounds required is unknown. Using the pre-grouping algorithm with a sufficient value (i.e., empirically 2E5 for normal plastome percent), five rounds of extension typically recruited target reads covering the whole plastome (although 15 rounds were required in a very few cases with extremely low plastome coverage (data not shown)).

The optimal Word size is dictated by read length, read quality, total number of reads, percentage of organelle DNA content, heterogeneity of organelle base coverage, and other factors. If there is no user-assigned Word size value, “get_organelle_from_reads.py” will automatically estimate an optimal Word size value using a set of customized empirical functions based on the characteristics of the dataset. The automatically estimated Word size tends to be small to enhance the success rate, but possibly at the cost of increasing the computational burden (Fig. 5). Importantly, though, the automatically estimated Word size does not guarantee the best performance in assembly results (see eight plastome samples with customized parameters in our tests) nor in computational costs.

### *k*-mer in GetOrganelle

GetOrganelle uses SPAdes as the core de novo assembler, which allows the user to use a *k*-mer gradient for assembly. One advantage of this is that SPAdes combines the assemblies from multiple *k-*mers. In our tested 50 plant datasets, the most successful assemblies were the results of the largest *k-*mers that the data permitted, which is 127 for read length ≥ 150 bp or 91 for read length up to 100 bp. Someone might draw a specious conclusion that the base coverage is usually high enough to use the largest *k*-mer for assembling the complete plastome or mitogenome from WGS data. However, except for those with low base coverage for the plastome (e.g., SRR5602610, ca. 14×), plastome assembly still suffered from the base coverage heterogeneity or read error, which create regions where large *k-*mers do not overlap. For example, if only one large *k-*mer value was used for each run (i.e., 91 for read length ca. 100 bp and 127 for read length ≥ 150 bp), some samples (tests not shown) would not assemble a complete circular plastome, while by using a *k-*mer gradient, those samples achieved a complete plastome at the same large *k-*mer.

Another advantage of using a *k*-mer gradient is that GetOrganelle could iteratively attempt to disentangle the assembly graph of each *k-*mer from the largest to the smallest, then find a larger one with the organelle-sufficient graph. A larger *k-*mer value is preferable when there are longer repeats and coverage is sufficiently high. However, when the largest *k-*mer used in the analysis does not obtain the complete circular plastome/mitogenome, the assembly graph of a smaller *k-*mer is checked automatically. The “GetOrganelle-auto” runs of SRR5602587 and SRR5028199 benefitted from this design.

### Computational consumption of GetOrganelle

Freudenthal et al. [24] showed that GetOrganelle has moderate efficiency in both time and memory usage among tested assemblers. However, they only used the default options for evaluation, which are designed to have high chloroplast genome completion rate, rather than high computational efficiency. If the aim of studies is not to assemble complete organelle genomes, researchers could easily adjust the options, such as increasing the Word size or turning on “--fast” to significantly speed up assembly and reduce memory usage, and still keep both the success rate and assembly quality at a higher level than those of other assemblers (additional tests via https://github.com/Kinggerm/GetOrganelleComparison version 1.1.1). However, we do not recommend these options. Firstly, in the current version of GetOrganelle, the complete circular assemblies are better than incomplete results, justifying a reasonable tradeoff of assembly accuracy against computation speed. Secondly, extremely high Word size may generate higher error rate in assemblies with two cases in the 50 plant datasets (i.e., GetOrganelle-W0.8 runs of SRR5602577 and SRR5602597).

## Conclusions

GetOrganelle is a fast and versatile toolkit for de novo assembly of complete and accurate organelle genomes using low coverage WGS data. Our evaluations show that the GetOrganelle toolkit can efficiently and accurately assemble different types of organelle genomes from a broad range of organisms. In general, compared with NOVOPlasty, GetOrganelle has far better success rates for assembling plastomes while consuming similar or even less computational resources. Additionally, GetOrganelle-reassembled plastomes generally have much higher accuracy than those reassembled by NOVOPlasty or published ones that were assembled by various tools in accordance with the read mapping evaluation. GetOrganelle can also generate all possible configurations when plastomes or mitogenomes have flip-flop configurations or other isomers mediated by repeats.

Potential applications of GetOrganelle include quickly extracting organelle genomes from whole genome assemblies and evaluating organelle genome quality. Assembling organelle genomes from metagenomic data would also be possible by using a customized database and scheme. The maximum extending length option enables rough control of the length of the target assembly, which could be used to quickly assemble interesting loci or genes from the metagenomic and transcriptomic data. Additionally, the Python Classes and Functions defined in GetOrganelleLib could be used to manipulate and disentangle non-organelle assembly graphs.

Currently, GetOrganelle exports all possible configurations without using library information of the paired-end reads. However, the long insert size library or long-read sequencing data can be used for repeat resolution and configuration verification. A function that could use this information and estimate the proportion of all the potential isomers (configurations) is expected in a future version of GetOrganelle. Improvements in the seed databases and the label databases are also expected, which should result in better parameter estimation and higher success rates in assembling mitogenomes.

## Methods

### Workflow of organelle genome assembly using GetOrganelle

GetOrganelle v1.6.2 consists of two major scripts (“get_organelle_from_reads.py” and “get_organelle_from_assembly.py”) and 17 minor scripts (under the directory “Utilities”; for processing or evaluating organelle assemblies) (Fig. 1), a series of libraries (under the directory “GetOrganelleLib”; including default seed database, default label database, and Python Classes/Functions), and dependencies (under the directory “GetOrganelleDep”; including Bowtie2, SPAdes, BLAST+). The major script “get_organelle_from_reads.py” could pipe all 5 steps described below (also see green solid arrows in Fig. 1) together using a single line command to assemble organelle genome(s) from raw reads. The major script “get_organelle_from_assembly.py” could extract organelle genome(s) from assembly graphs generated using SPAdes [15] or Velvet [54] (steps 4–5; also see blue solid arrows in Fig. 1).

#### Step 1. Mapping reads to seed and assembling seed-mapped reads for parameter estimation

The initial step of assembling target organelle genome(s) from reads via GetOrganelle (“get_organelle_from_reads.py”) uses Bowtie2 to map reads to seed sequence(s) (i.e., the default seed database), which may include complete reference organelle genome(s) or organelle fragment(s) (Fig. 1, green solid arrow 1). Currently, the default seed of GetOrganelle (under the directory “GetOrganelleLib/SeedDatabase”) covers embryophyte plastomes, non-embryophyte plastomes, embryophyte mitogenomes, embryophyte nuclear ribosomal DNA, animal mitogenomes, and fungal mitogenomes (in the option referred as “embplant_pt,” “other_pt,” “embplant_mt,” “embplant_nr,” “animal_mt,” “fungus_mt,” respectively). These mapped reads are here called seed-mapped reads (stored at *.initial.fq).

The seed-mapped reads will be treated as initial “baits” to recruit more target-associated reads in the next step. In the “auto” mode (see the last paragraph of step 2 below), the seed-mapped reads will be also coarsely assembled into seed contigs, which will be used for parameter estimation in step 2.

#### Step 2. Recruiting more target-associated reads through extending iterations

After creating “baits,” GetOrganelle (“get_organelle_from_reads.py”) recruits new target-associated reads by comparing candidate reads to “baits” and updating the “baits” with overlapped new reads (Fig. 1, green solid arrow 2). In this extension process, the key comparison method for determining overlaps is classic substring hashing. Substring(s) are referred as Word(s) here to distinguish them from *k*-mers, a similar concept in the assembly process. The uniform length of the Words is thus named as Word size.

Before the core extension iterations, “get_organelle_from_reads.py” creates an index and assigns unique ids for each set of duplicated reads to avoid repeatedly calculating (information stored at file “temp.indices.1”). Those reads with duplicates can also be used for downstream “pre-grouping”. Pre-grouping is an algorithm for speeding up target-read recruitment. This algorithm is based on the idea that it would be more efficient to firstly compare reads that are more likely to be target-associated. Given that the organelle genomes usually have more copies, and hence higher base coverage than most non-organelle chromosomes, the duplicated reads are more likely to be organelle-associated than non-duplicated reads. The “get_organelle_from_reads.py” script will group a certain number of duplicated reads (after option “-P”) into groups based on read overlap using the same substring hashing method mentioned above. Any groups, including those with only a single read, will have a hash table storing Words chopped from all reads of this group. Any two groups sharing at least a single Word in their hash table will be merged. After pre-grouping, a group resembles a set of connected pseudo-contigs (information stored at file “temp.indices.2”). During the following extension iterations, once a read is accepted as a target-associated read, all other reads (ids) in the same group will be marked as acceptable.

The “get_organelle_from_reads.py” script begins the core extension iterations with constructing a hash table, by cutting the initial reads (“baits”) into Words and adding those initial Words to the hash table (AW). If a Word of a candidate read hits the AW, all Words of this read are also added to AW, and the index of this read will be marked as accepted (Accepted Index, AI). As mentioned above, all other read ids in the same group will be treated as accepted. During each iteration (round), “get_organelle_from_reads.py” goes through all candidate reads one by one to check whether a read is acceptable. After a user-specified number of rounds or when no new read has been accepted in a complete round, “get_organelle_from_reads.py” will stop the extension process and output all accepted reads (stored at file “filtered_*.fq”). For low memory machines or testing purposes, accepted reads per round can be outputted separately after each round (followed with flag “--out-per-round”) along with AW emptied after each round.

Word size (followed with flag “-w”), like *k-*mer length in assembly, is crucial to the feasibility and efficiency of this process. The best Word size for extension is affected by read length, read quality, total number of reads, percentage of organelle genome reads, heterogeneity of organelle base coverage, and other factors. In the “auto” mode when there is no user-assigned Word size value, “get_organelle_from_reads.py” will automatically estimate a proper Word size with a set of empirically customized functions, based on the data characteristics.

#### Step 3. Conducting de novo assembly

The recruited target-associated reads will be then automatically assembled using SPAdes (Fig. 1, green solid arrow 3). Both paired and unpaired reads will be used. The outputs of each *k-*mer of SPAdes include an assembly graph (FASTG format), which records the connections of contigs as a graph with some allelic polymorphism and assembly uncertainty. Other assemblers that are able to generate the assembly graph, such as Velvet, may be used for completing this step, but are not yet implemented in GetOrganelle. The intermediate results are stored in the subfolder “filtered_spades”.

#### Step 4. Roughly filtering for target-like contigs

Because sequences are often shared among plastomes, mitogenomes, and nuclear genomes, the accepted reads from step 2 sometimes unavoidably include non-target reads. As a consequence, the output assembly graph might also include non-target contigs. However, previously reported tools did not account for or adequately addressed this concern.

GetOrganelle searches for the target-like contigs from the original assembly graph file by jointly using the contig label table, contig connections, and contig coverages (Fig. 1, green solid arrow 4 and blue solid arrow 1). To create the contig label table, GetOrganelle takes the contigs in the assembly graph as the queries, conducts the BLAST search against a local label database (see next paragraph), generates the BLAST hit table, and converts the generated BLAST hit table into the contig label table, which records the gene identities and organelle types of those BLAST matches. By conservatively deleting non-target contigs, GetOrganelle outputs a simplified assembly graph file, along with a concomitant cognominal TAB-formatted file recording the contig label table (with the extension “.csv” to be in conformity with Bandage). This step is completed automatically by the two major scripts or can be independently executed using the script “slim_fastg.py”.

For GetOrganelle, the default label database of a certain organelle is made from the coding regions of that organelle genome. We created six default label databases that correspond to the six types of organelle genome in the seed databases. A contig that hit the target organelle database will be labeled with gene identity in the “.csv” file and called target-hit-contig here. Any contig that is directly or indirectly connecting to that target-hit-contig is called a target-associated-contig. Here, we define a group of interconnected contigs as a connected component of the assembly graph. GetOrganelle by default retains all connected components with target-hit-contig(s). Additionally, in the embplant_pt or embplant_mt mode, GetOrganelle by default retains both plastome and mitogenome connected components for downstream clustering contigs by coverage. Generally, this roughly filtering step is designed to be conserved and avoiding removing true target contigs.

#### Step 5. Identifying target contigs and exporting all configurations

GetOrganelle then uses the simplified assembly graph file and the contig label table to (5a) further accurately identify (narrow down to) target organelle contigs, (5b) estimate multiplicities (copy number) of contigs in an organelle-only graph, and (5c) export all possible distinctive path(s) [stored as FASTA file(s)] from the organelle assembly graph (stored as a cognominal GFA format file) (Fig. 1, green solid arrow 5 and blue solid arrow 2). Each path represents a possible configuration of the target organelle genome. This step is fulfilled by the two major scripts or can be separately executed using the script “disentangle_organelle_assembly.py”. In case of organelle genome with a large number of repeats, GetOrganelle sets up an option for limiting the calculation time of disentangling to avoid generating inexhaustible combinations. When the major scripts failed to export circular sequence(s) from the assembly graph for the reasons enumerated below in step 5′ or because of the time limit, they will execute a second run to export the contigs, which would be mainly the target-hit-contigs.

Three concepts need to be clarified:

1. An “organelle-sufficient graph” is an assembly graph with contigs completely covers one complete organelle genome.
2. An “organelle-only graph” is an assembly graph only with true contigs of one organelle genome.
3. An “organelle-equivalent graph” is both an organelle-sufficient graph and an organelle-only graph.

GetOrganelle requires three assumptions to disentangle the assembly and declare the result as a complete circular organelle:

- *Assumption 1*: All configurations, if there are more than two, of the target organelle genome are compositionally identical. This assumption limits the multiplicities of contigs to be the same among different configurations. In other words, polymers are found in real plastid DNA molecules [55], whereas GetOrganelle can only export the monomer form; potential sub-genomic configurations are currently not implemented in the current version. If there are parallel contigs caused by nucleotide polymorphism, all subgraphs composed of any of those polymorphisms will be disentangled independently. Therefore, all configurations of each subgraph will be compositionally identical.
- *Assumption 2*: The topology of each organelle genome will be represented as a single circular molecule. This assumption holds when the real organelle genome is a circular molecule or organized in polymers (most plastomes, and type I and type II mitogenomes) and the assembly graph is an organelle-sufficient graph. If this assumption is violated, GetOrganelle only exports the target contigs.
- *Assumption 3*: The coverage values of contigs of the same organelle genome are generally proportional to their multiplicities (copy numbers). Therefore, coverage values of contigs with the same multiplicity of the same organelle genome generally are consistent.

##### 5a. Further identifying target organelle contigs

Using the BLAST hit information alone to identify target organelle contigs is risky. Some contigs, including mitochondrial contigs that have short sequence of plastome origin or target-like shallow-depth contaminant contigs, would be labeled incorrectly as target-hit-contigs (false positive). On the other hand, some sequences might be true target contigs but are too short or divergent from sequences in the label database to be labeled as target-hit-contigs (false negative). Therefore, we used additional information to improve the identification of target contigs, such as the assembly graph characters (Assumptions 1 and 2) and contig coverage values (Assumption 3). GetOrganelle uses an integrated strategy that iteratively uses all or part of the following modules to approach this task until no more changes are going to be made to the assembly graph.

1. Using the BLAST hit information to roughly cluster contigs with target labels. Specifically, GetOrganelle first calculates a customized hit weight value (HW) for each BLAST hit record (gene identity label restored from the contig label table). For records representing the same gene in the local BLAST database, only the record with the best HW is kept as the only valid hitting record for that gene. The HW of a hit record is simply defined as the product of the hitting length of the query (HL) and query contig coverage (QC) (HW = HL * QC). Given our experience that the false positive hits generally correspond to shorter length and shallower depth contigs, HW can be a criterion for excluding the false positive hit records. Each gene in the BLAST database is thus aligned to no more than one contig in the assembly graph. Then, GetOrganelle calculates a customized contig weight value (CW) of a target organelle genome for each contig as CW_target_ =  ∑ HW_target_. For example, the plastid CW for a contig is defined as the sum of the HWs of all plastid gene hit records of that contig, while the mitochondrial CW for the same contig is defined as the sum of the HWs of all mitochondrial gene hit records of the same contig. For a contig, if the target CW is much larger (default factor: 3 times) than the non-target organelle CW, this contig would be labeled as a target-anchor contig (very likely to be a true target contig), and vice versa. Using the HW and CW, GetOrganelle roughly eliminates most non-target contigs with false positive BLAST hits.
2. Adding more target labels to some target contigs that do not hit the label database according to assembly graph characters. Based on Assumption 2, any configuration of the target organelle genome is a single circular molecule. As a result, in an organelle-sufficient graph, both ends of any true target contig should be connected to at least one true target contig. If the tail end of a true target contig, Contig A (marked as A_tail_) has only one edge that connects A_tail_ and the head end of another unknown contig, Contig B (B_head_), then Contig B should be a true target contig. However, in a real assembly graph with missing contigs (incomplete organelle genome), Contig B may be missing and the unknown contig connected to A_tail_ may be a non-target contig. In consideration of complicated situations like this, only when Contig B is between two target-anchor contigs (Contig A and Contig C) with the sequence (A_tail_-B_head_-B_tail_-C_head_), and when A_tail_ only connects to B_head_ and C_head_ only connects to B_tail_, GetOrganelle labels Contig B as a target-anchor contig with CW = 0.
3. Using coverage values of contigs to remove contigs with coverage value that significantly deviates from the target-anchor contigs. Based on Assumption 3, GetOrganelle uses the Gaussian mixture distribution to approximate the coverage values of all contigs in the simplified assembly graph, which is a mixture of different organelle contigs and nuclear contigs. In most cases of empirical plant genome skimming data, the plastome has significantly higher coverage than the mitogenome, the coverage of which in turn is higher than the nuclear genome except for highly repeated regions. Therefore, in a plant WGS dataset, the coverage values of plastid and mitochondrial and nuclear contigs are expected to be classified into different Gaussian components of the Gaussian mixture distribution. GetOrganelle could thus delete the contigs with coverage value far from the target coverage distribution. Specifically, GetOrganelle applies an EM (Expectation-Maximization) algorithm with the semi-supervised learning and the weighted Gaussian mixture model to cluster the coverage values of all candidate contigs. Here, the semi-supervised learning means that the coverage values of the target-anchor contigs (the labeled data) are not updated during EM iterations. The coverage value of a contig in the Gaussian mixture model is weighted by the length of the contig.
4. Removing contigs isolated from the main target connected component that includes the target-anchor contigs. Based on Assumption 2, true target contigs in an organelle-sufficient assembly graph should occur in one connected component. Thus, for a real organelle-sufficient assembly graph, GetOrganelle retains the connected component with the most target-anchor contigs and deletes other such connected components. Specifically, GetOrganelle calculates a customized target weight value (TW) for each connected component of the assembly graph. The TW of a connected component is defined as the sum of the target CWs of all contigs in that connected component. Assuming organelle-sufficiency, GetOrganelle sorts connected components by their TWs, finds the connected component with the significantly largest TW (100 times larger than the second largest TW by default), and removes the contigs of other connected components from the assembly graph. If GetOrganelle fails to find such a connected component, disentangling the assembly graph as a circular organelle genome fails and GetOrganelle reverts to “linear mode.” In a “linear mode”, when GetOrganelle tries to disentangle the assembly graph as contigs or scaffolds, several connected components with large TW are retained, and only the connected components with TWs 10,000 times (by default) less than the largest TW will be removed.
5. Removing tip contigs. A tip contig is a contig with one or both ends that do not connect to any other contigs in the assembly graph nor to itself as circular. Based on Assumption 2, an organelle-equivalent graph will not contain any tip contig, because any partial sequence of a circular DNA molecule will have both upstream and downstream sequences. GetOrganelle will check whether a tip contig is a target-anchor contig before removing it. If the tip contig is a target-anchor contig, it is likely that Assumption 2 is violated, and in most cases, the assembly graph is not an organelle-sufficient graph but rather “a broken organelle graph”.

##### 5b. Estimating the multiplicities of contigs in an organelle-only graph

There are sources of information resources for estimating multiplicities for contigs in GetOrganelle. One is contig coverage value (*k*-mer coverage in practice but referred to as coverage in this paragraph). According to Assumption 3, we could roughly estimate the multiplicity of each contig based on its coverage. Given that any contig in an organelle-only graph would have at least one copy, GetOrganelle first assumes all contigs have the multiplicity of one and estimates a primary value of the average coverage of the target genome. Then, GetOrganelle optimizes the multiplicities of all contigs by dividing the coverage value of a contig by the average coverage and rounding the result to the nearest integer. GetOrganelle iteratively optimizes the average coverage of the target genome and the multiplicities of the contigs, with the constraint that the genome average coverage should not be below the minimum coverage of all contigs according to Assumption 1. Here, the stabilized multiplicities estimated on the base of the coverage values of contigs are called observed multiplicities (O_C*i*_, with _C*i*_ denoting the contig name) (Fig. 2).

Another type of information comes from the graph characteristics, which offers a set of hard constraints for the multiplicities of contigs (M_C*i*_, with _C*i*_ denoting the contig name). Using the Python library Sympy, GetOrganelle creates a set of linear equations to characterize the multiplicity relationship among connected contigs. In detail, there are mainly four constraints to build this set of equations. First, the multiplicity of a self-loop contig has no constraints. Second, if Contig A is not a tip contig nor a self-loop contig, M_CA_ is equal to the sum of the multiplicities of the contigs connected to A_head_ and equal to the sum of the multiplicities of the contigs connected to A_tail_. Third, the multiplicity of a tip contig is arbitrarily set to 1 to avoid over-estimation, although this risks failure in solving the equations. Last, in considering symmetry of large inverted repeats (such as IRs in plastome), the multiplicity of a sequential repeat contig is constrained to be an integer multiple of the multiplicity of one of its nearby contigs, e.g., the multiplicity of a sequential repeat inside the IR region of a plastome assembly graph must be an even number and no smaller than 4. This set of equations would be then simplified using Sympy.solve. The multiplicity values of all contigs would be represented as linear expression of several free variables.

By minimizing the difference of the multiplicities based on the above two types of information using least square, meaning minimizing ${\sum}_{i=0}^{\mathrm{n}}{\left({\mathrm{M}}_{\mathrm{C}i}-{\mathrm{O}}_{\mathrm{C}i}\right)}^2$, GetOrganelle achieved the values of those freedom variables, therefore all the M_C*i*_ (Fig. 2).

##### 5c. Exporting all possible distinctive path(s)

GetOrganelle then exhaustively searches for all possible paths from this organelle-only graph with contig multiplicities. Each configuration combination would be saved as an independent FASTA file, with the same sequence-name style to manual completion using Bandage [30] (see below). A circular sequence would also be marked “(circular)” in the sequence name. For plastomes with repeats inside the large IRs, there would be 6 paths, meaning 6 potential isomers (see Fig. 2; another similar but more complicated example is SRR5602601 with 12 paths). However, in considering symmetry, only those isomers with identical large IRs (path1 & 2 in Fig. 2) are biologically possible paths. In these cases, GetOrganelle would mark these results with identical IRs as the first repeat pattern in the file name.

#### Step 5′. Manual completion

If GetOrganelle fails to export a complete circular organelle genome, because of insufficient target assembly graph, too short a disentangling run time, too many possible configuration sequences, or misidentification of target contigs, manual completion is needed to clean “noisy” and non-target contig/scaffold connections (Fig. 1, gray solid arrow 4 and 5). The simplified assembly graph can be visualized using Bandage [30]. Meanwhile, the concomitant annotation “csv” file can be imported into Bandage and added to the graph as labels, which helps to manually identify target-like contigs/scaffolds. The semi-manually cleaned assembly graph can be then finalized using another main script “get_organelle_from_assembly.py” (Fig. 1) or be manually exported from the cleaned assembly graph using Bandage.

### Assessing the performance characteristics of GetOrganelle

To investigate the performance characteristics of GetOrganelle, we varied relevant parameters of the program: word size values (as the form of WSR, i.e., 0.3, 0.35, 0.4, 0.45, 0.5, 0.55, 0.6, 0.65, 0.7, 0.75, 0.8, 0.85, 0.9), the number of rounds (unlimited vs. minimum), and pre-grouping either activated (-P 2E5, i.e., using the top 2E5 duplicated reads to conduct the pre-grouping) or disabled (-P 0). The minimum number of rounds mode is defined as fewest rounds of extension iterations required to obtain a complete plastome or stabilizing an incomplete plastome.

We used these various settings to assemble the plastome of the angiosperm species *Haberlea rhodopensis* Friv. (Gesneriaceae) from a reduced published dataset (500 Mb, SRA: SRR4428742), with the complete plastome of a gymnosperm species, *Gnetum parvifolium* (Warb.) W.C.Cheng (GBK: NC_011942.1) as the seed. We additionally use only the *rbcL* gene sequence of *Gnetum parvifolium* (GBK: NC_011942.1) as the seed and the WSR of 0.75 to assemble the same dataset.

To assess the characteristics of recruited reads per round, the option “--out-per-round” was specified for the script “get_organelle_from_reads.py”. The script “round_statistics.py” was used to assess the increasing cover percent of the organelle genome using a read mapping approach. Using Bowtie2, this script maps reads of each round to the final assembled plastomes and calculates the percentage of bases in the plastome that are covered by mapped reads over a certain coverage threshold (the defaults were 0 and 10). The minimum number of rounds occurs when the percentage of covered bases reaches 100% or stays unchanged and when the final assembly is stabilized. Besides, if Python library Matplotlib is available, the script “round_statistics.py” could generate the base coverage across the organelle genome plot for each extension round to visualize the extension process.

### De novo assembly of the plastomes of 50 plant datasets using GetOrganelle and NOVOPlasty

To evaluate the working efficiency and assembly success, we selected 50 datasets of vascular plants with raw reads from the GenBank Sequence Reads Archive (SRA) (Additional file 2: Table S1). The 50 vascular plants represented 42 species of angiosperms (from eight major clades, 21 orders and 29 families), four species of gymnosperms, three species of ferns, and one species of lycophytes. Notably, the raw reads of these 50 samples are associated with published plastomes [56–59], allowing comparison with newly reassembled plastome using GetOrganelle. Since 2018, NOVOPlasty has received more than 400 citations for assembly chloroplast genome in Google Scholar (accessed 31 Dec 2019) and became one of the most widely used tools for plastome assembly. We thus reassembled 50 samples using NOVOPlasty for comparisons.

The data resources are paired-end reads. The read length varied from 100 to 300 bp (Additional file 2: Table S1). In all tests, if the tested data included fewer than 10,000,000 reads for each end, we used all the reads; if the data included more than 10,000,000 reads of each end, we only select the first 10,000,000 reads for each end. We set up four testing groups, i.e., three groups with different word size values (w = 0.6, 0.7, 0.8) (i.e., GetOrganelle-W0.6, GetOrganelle-W0.7, GetOrganelle-W0.8) and an auto-estimated word size group (i.e., GetOrganelle-auto). The extension rounds of all tests were set to 10. All other options including the seed were set to default. Because incomplete assemblies are unsuitable for comparing mapping qualities in the next part, we additionally added extra runs for eight samples, in which GetOrganelle-auto could not achieve complete plastomes, with customized options (GetOrganelle-customized) for mapping quality comparison. A detailed record of commands, as well as the final results and log files recording the memory usage and time cost of all the tests are available at https://github.com/Kinggerm/GetOrganelleComparison (version 1.1.1).

Plastomes from the same 50 datasets were also reassembled by NOVOPlasty using four *k-*mer values, i.e., 23, 31, 39, and 47. The config file of NOVOPlasty was downloaded from the NOVOPlasty GitHub repository (https://github.com/ndierckx/NOVOPlasty/blob/master/config.txt), with “Type” as “chloro,” “Genome Range” as 15,000–180,000, “Save assembled reads” as “yes,” “Seed Input” as the same seed as running GetOrganelle, and “Read Length” as the mean read length of each sample (seed Additional file 2), with all other parameters unchanged.

### Read mapping to evaluate plastome assemblies

The script “evaluate_assembly_using_mapping.py” was used to evaluate circular/non-circular assemblies (Fig. 1, gray solid arrow 8). It uses Bowtie2 to map reads to circular/non-circular assemblies; parses the SAM file; counts the number of mapped paired and unpaired reads; counts matched bases for each site (M), mismatched bases for each site (X), insertions between any two sites (I), and deletions for each site (D); and calculates the average and standard deviation of matched depth (ΣM and var(M)), average and standard deviation of mismatched depth (ΣX and var(X)), average and standard deviation of insertions between any two reference sites (ΣI and var(I)), and average and standard deviation of deletions per reference site (ΣD and var(D)) for each contig and the whole assembly. If ΣM > 0, a customized error rate would be also calculated as (ΣX + ΣI + ΣD)/ΣM with a customized deviation as (var(X) + var(I) + var(D))/ΣM. If “--draw” is chosen, the script “evaluate_assembly_using_mapping.py” generates the plot of M/X/I/D at each site/site-interval across the whole assembly using Python library Matplotlib. For reproducibility when using the same random seed, a circular assembled sequence was relinearized to make sure that biologically the same circular plastome would have identical start and end since identical linear sequence. By default, Bowtie2 does not support mapping reads to a circular sequence, the script “evaluate_assembly_using_mapping.py” gets around this problem by adding an extra fragment of the head of the original sequence to the tail, and counting the mapping statistics (M/X/I/D) of the sites in the extra fragment back to the statistics of the head part of the original sequence.

The script “evaluate_assembly_using_mapping.py” was used to assess all the assembled plastomes. The abnormal characters, such as “*” and “-” in the FASTA-format assemblies of NOVOPlasty, were replaced with “N.” NOVOPlasty would also produce multiple completely different sequences with the same sequence name in the assemblies, which would be also modified with different names before assessment. For NOVOPlasty, the evaluation statistics of each sample were based on the best result among those using different *k-*mer values. Here the best result is determined in turn by being true circularized, the largest number of mapped paired reads, the largest number of mapped unpaired reads, the largest matched depth, the smallest error rate, the smallest deviation of matched depth, and the smallest deviation of error rate. For GetOrganelle, except for 8 samples that were generated using the customized parameters, other evaluation statistics were based on the assemblies from the “GetOrganelle-auto” runs.

For each sample, when comparing the evaluation statistics of different assemblies, the “best” mapped reads (the hat mark in Table S3) is defined as the largest number of mapped paired reads or equal-largest number of mapped paired reads with the largest number of mapped unpaired reads; the “best” mapped depth is defined as the largest matched depth or equal-largest matched depth with the smallest deviation of matched depth; the “best” error rate is defined as the smallest error rate or equal-smallest error rate with the smallest deviation of error rate.

### De novo assembly and evaluation of the mitogenomes using GetOrganelle and NOVOPlasty

In total, 56 animal (the test samples of MitoZ [14], see Additional file 2: Table S3) and 50 fungal samples (Additional file 2: Table S4) were used to test the capability of GetOrganelle to assemble mitogenomes. The mitogenomes of plants generally have a relatively slow nucleotide substitution rate [9, 60]; therefore, it is feasible for GetOrganelle to achieve sufficient reads to construct the mitogenome-sufficient assembly graph even with a remotely related seed, provided that there is enough coverage. However, there are two main challenges for short-read sequencing data to confirm the mitogenome architecture. Firstly, lots of repeats in the plant mitogenome cause the awkward tangles in the assembly graph [47, 61, 62]. Secondly, most of plant mitogenomes are not one single circular structure; rather they often consist of one large circular molecule and small circular plasmid-like molecules (type III), or homogenous linear molecules (type V) [63]. There are also frequent horizontal transfers from plastome to mitogenome. In this case, when the coverage of the plastome is much higher than that of mitogenome, the multiplicity of shared contigs would be hard to estimate. When the coverage of the plastome is similar to that of the mitogenome, the parallel contigs in between the shared contigs would be difficult to distinguish. Thus, we did not include plant mitogenome testing due to the general infeasibility of assembling complete circularized plant mitogenome from WGS data.

The data resources are paired-end reads. The read length varied from 92 to 301 bp (see Additional file 2: Table S3, Table S4). In animal tests, if the tested data included fewer than 75,000,000 reads for each end, we used all the reads; if the data was more than 75,000,000 reads of each end, we only used the first 75,000,000 reads for each end. The same maximum number of reads for fungi was 15,000,000. All GetOrganelle tests were performed with default settings with the *k-*mer values set to 21,43,65,87,127. All NOVOPlasty test were performed with the default *k*-mer 39 (also been the best *k*-mer in the plastome analysis). Commands, results, and log files of all the tests are available at https://github.com/Kinggerm/GetOrganelleComparison (version 1.1.1).

The script “slim_fastg.py” was used to evaluate mitogenome assemblies by generating the gene hits table. “slim_fastg.py” could conduct a BLAST search against the label database and generate a concomitant cognominal TAB-formatted file recording many hitting information of contigs. By summarizing the gene hits of each contig, we calculated the number of gene hits for each assembly.

### Computer resources for testing

All assemblies were executed on an Intel Xeon CPU machine containing 144 cores of 2.40 GHz, a total of 3 TB of RAM (64 GB RAM is sufficient for all tests, while the 3 TB RAM makes it possible to run different tests simultaneously), and set up with Linux (Linux version 3.10.0-514.el7.x86 (Red Hat 4.8.5-11)). The testing environment also includes Python v3.6.5, Perl v5.16.3, GetOrganelle v1.6.2, Bowtie2 v2.3.5.1, SPAdes v3.13.0, BLAST 2.2.30+, Numpy v1.13.3, Scipy v0.19.1, Sympy v1.1.1, Matplotlib v3.0.2, Psutil v5.4.7, and NOVOPlasty 2.7.2 [23]. GetOrganelle is capable of multi-processing in mapping and assembly process, which was disabled in this test for comparable with NOVOPlasty. Tests can be reproduced by accessing zenodo (doi: 10.5281/zenodo.3943877) or Github (https://github.org/Kinggerm/GetOrganelleComparison version 1.1.1).

## Supplementary information

**Additional file 1: Figure S1.** Reads mapping comparisons among the plastomes reassembled using GetOrganelle and NOVOPlasty, and the plastome from GenBank (KY085912) based on the same raw data SRR5602602 (*Laurus nobilis* L.). **Figure S2.** The covering positions and corresponding coverages at the plastome of newly captured reads during each round using GetOrganelle, with different arguments. A whole plastome and an *rbcL* region (right) from a gymnosperm species *Gnetum parvifolium* (Warb.) W.C.Cheng (GenBank nucleotide accession number: NC_011942.1) as the seed to assemble the plastome of an angiosperm species *Haberlea rhodopensis* from an online WGS dataset (GenBank SRA accession number: SRR4428742).

**Additional file 2: Table S1.** The sampling and basic assembly information of 50 plant samples. **Table S2.** Evaluation of the published plastomes, GetOrganelle reassembled plastomes and NOVOPlasty reassembled plastomes of same GenBank SRA accession number using reads mapping. **Table S3.** The sampling and basic assembly information of 56 animal samples using GetOrganelle and NOVOPlasty. **Table S4.** The sampling and basic assembly information of 50 fungi samples using GetOrganelle and NOVOPlasty. **Table S5.** Performance characteristics of GetOrganelle using the plastome of a gymnosperm species Gnetum parvifolium (Warb.) W.C.Cheng (GenBank SRA accession number: NC_011942.1) as the seed to assemble the plastome of an angiosperm species Haberlea rhodopensis from an online WGS dataset (GenBank SRA accession number: SRR4428742).

**Additional file 3.** Review history.

## Data Availability

Project name: GetOrganelle Project home page: https://github.com/Kinggerm/GetOrganelle Archived version: 1.6.2 [64] Operating system: Linux/MacOS Programming language: Python 2.7.11 or higher, or 3.5.1 or higher Other requirements: spades >=3.9, bowtie2 >=2.3, blast >=2.3, numpy >=1.16.4, scipy >=1.3.0, sympy >=1.4 License: GPL-3 See Additional file 2: Table S1, S3, & S4 for publicly archived datasets analyzed in this study. The commands and results of all analysis are available in zenodo via doi: 10.5281/zenodo.3943877 [65], also in https://github.com/Kinggerm/GetOrganelleComparison (version 1.1.1).
