# Supplementary material for: GetOrganelle: a fast and versatile toolkit for accurate de novo assembly of organelle genomes
Source: Genome Biol. 2020 Sep 10;21:241. doi: 10.1186/s13059-020-02154-5 (PMC7488116; doi:10.1186/s13059-020-02154-5)
Supplement: Supplementary file 1 — Additional file 1: Figure S1. Reads mapping comparisons among the plastomes reassembled using GetOrganelle and NOVOPlasty, and the plastome from GenBank (KY085912) based on the same raw data SRR5602602 (Laurus nobilis L.). Figure S2. The covering positions and corresponding coverages at the plastome of newly captured reads during each round using GetOrganelle, with different arguments. A whole plastome and an rbcL region (right) from a gymnosperm species Gnetum parvifolium (Warb.) W.C.Cheng (GenBank nucleotide accession number: NC_011942.1) as the seed to assemble the plastome of an angiosperm species Haberlea rhodopensis from an online WGS dataset (GenBank SRA accession number: SRR4428742). [file 13059_2020_2154_MOESM1_ESM.docx]

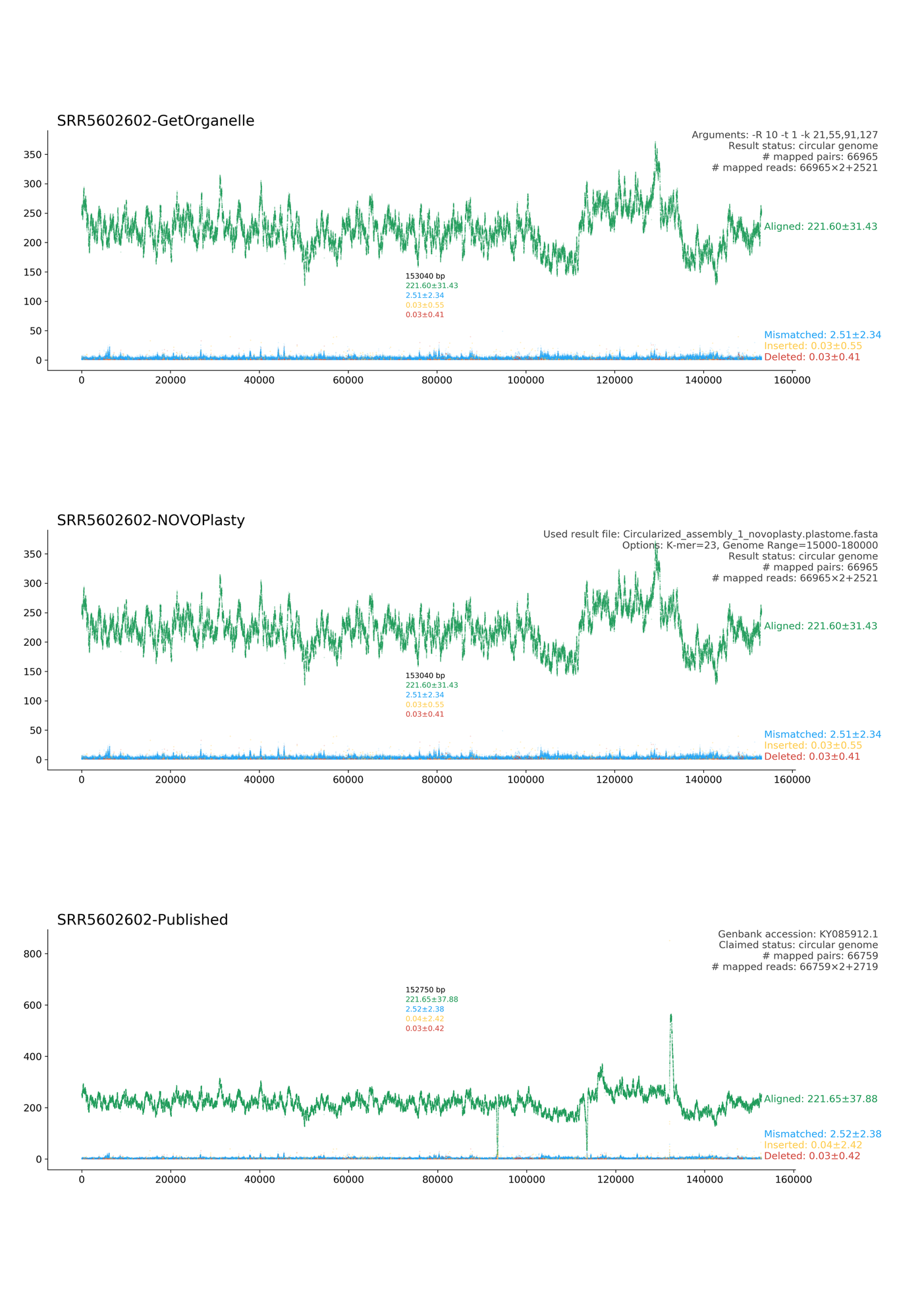


**Additional file 1: Figure S1.** Reads mapping comparisons among the plastomes reassembled using GetOrganelle and NOVOPlasty, and the plastome from GenBank (KY085912) based on the same raw data SRR5602602 (*Laurus nobilis* L.)


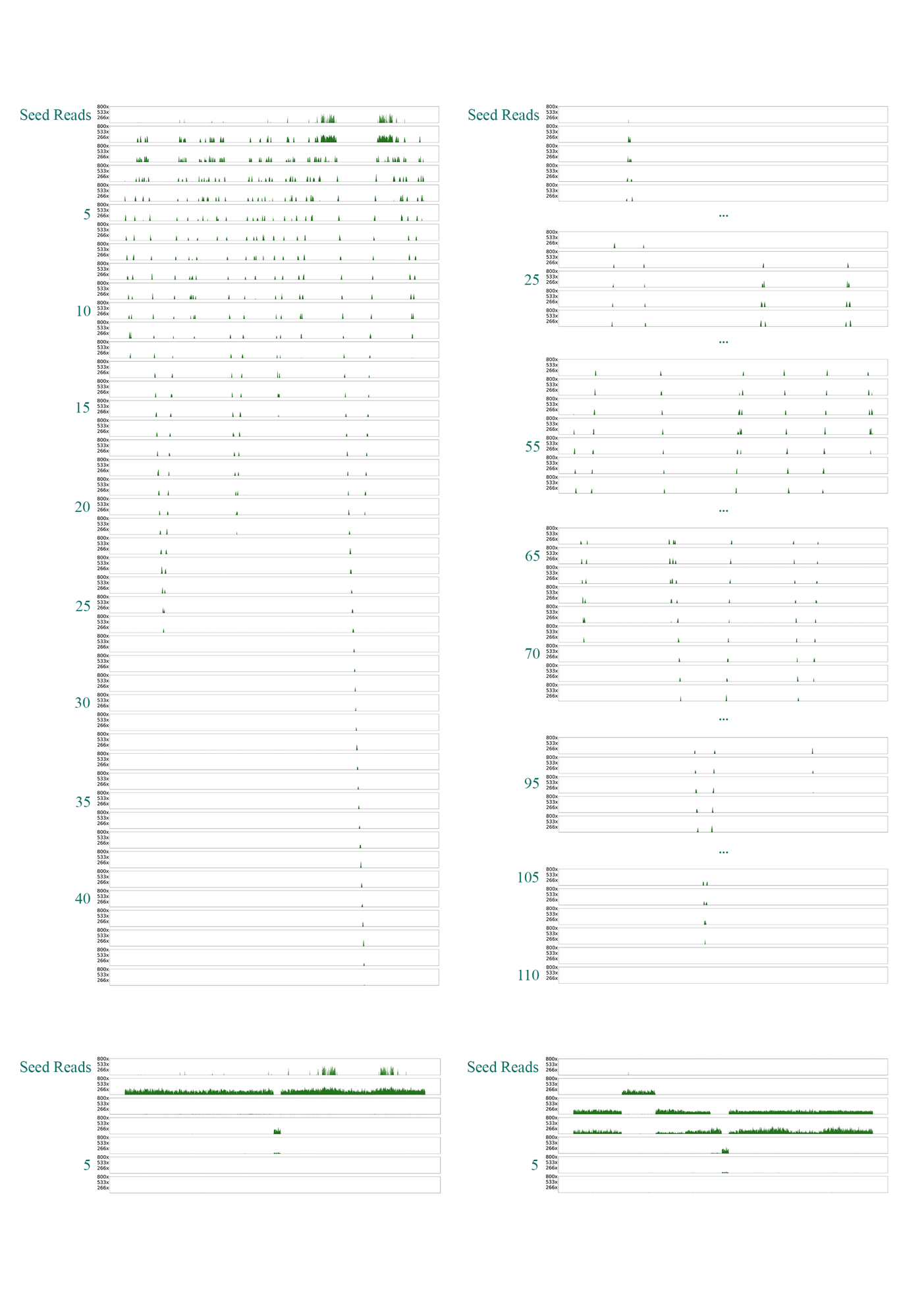
**Additional file 1: Figure S2.** The covering positions and corresponding coverages at the plastome of newly captured reads during each round using GetOrganelle, with different arguments. A whole plastome and an *rbcL* region (right) from a gymnosperm species *Gnetum parvifolium* (Warb.) W.C.Cheng (GenBank nucleotide accession number: NC_011942.1) as the seed to assemble the plastome of an angiosperm species *Haberlea rhodopensis* from an online WGS dataset (GenBank SRA accession number: SRR4428742).

word size: 0.75, pre-grouping: 200,000

word size: 0.75, pre-grouping disabled
