# Supplementary material for: GetOrganelle: a fast and versatile toolkit for accurate de novo assembly of organelle genomes
Source: Genome Biol. 2020 Sep 10;21:241. doi: 10.1186/s13059-020-02154-5 (PMC7488116; doi:10.1186/s13059-020-02154-5)
Supplement: Supplementary file 3 — Additional file 3. Review history. [file 13059_2020_2154_MOESM3_ESM.docx]

**Review history**

**First round of review**

**Reviewer 1**

Dear Colleagues,

I read you manuscript on GetOrganelle with great interest. Although slightly neglected by many genomics labs, the plastome is a crucial element of molecular biology, and should not be discarded with missassembled reads.

You wrote an excellent and convincing paper, which is very clear and organised. It also helps if you can already cite a successful third-party benchmark in your introduction! The discussion is very complete and thoughtful. The code for both the method and the figures are freely available on GitHub.

My only minor comments is that Figure 1, although very creative in its use of graphics, was rather overwhelming to me. In particular, I did not understand how the grey flow fits in the process. Reading through the text, I did not understand why the search for target-like contigs was mapped to green solid arrow 4 and blue solid arrow 2, when these two arrows are different in the figure. Also, the emoticons, although very charming, did confuse me out of context.

I successfully installed the dataset (using pip), but I did not have a test dataset at hand so was not able to test the software completely. Would it be possible for you to include a small test dataset in the installation?

Best regards,

Daniel Zerbino

**Reviewer 2**

In this study, the authors present a software tool called GetOrganelle that can be used to assemble plastid and mitochondrial genomes. This tool uses five steps, involving the 1) filtration of the read data set to keep reads that match to a seed set, 2) extension to identify additional likely reads, 3) de novo assembly of filtered reads, 4) removal of non-target contigs from the assembly, and 5) resolution of repeats and final assembly. Importantly, the authors evaluated the accuracy of assemblies from GetOrganelle and another popular tool (NOVOPlasty), a step that is very often ignored in published organellar genome studies, leading to an abundance of poor-quality genomes in the sequence databases.

Overall, the results are generally persuasive in demonstrating that GetOrganelle works well for plastid genomes. 44 of 50 plastomes agreed very closely or exactly with published sequences. Importantly, multiple reanalyses with different variations of parameters generally produced identical, or nearly identical, plastomes. Using multiple parameter combinations is a procedure I routinely use to verify my own plastome assemblies. The error and coverage analyses persuasively demonstrate that GetOrganelle's assemblies are probably more accurate than many published sequences, which indicate that many published plastomes likely have small errors, and some have major errors. This is an important observation that I have also routinely noted in my own reanalyses of data sets. There is certainly a need for a reliable automated approach for plastome assembly. The other softwared are very much inadequate, and very problematic for the field because they can produce junk assemblies.

1. One point of contention is that the authors seemed to have intentionally avoided some of the more difficult plastomes, such as in Ericaceae, Geraniaceae, and Pinus, which as the authors point out have an abundance of smaller repeats. Similarly, I don't think the authors tested any plastomes carrying insertions of mitochondrial DNA, such as carrot, milkweed, and bamboo plastomes. I imagine that these plastomes will be problematic for GetOrganelle (and almost certainly for other automated assemblers), but it would have been informative to see how GetOrganelle performs in these instances. At the very least, the authors should comment on the likelihood of success for plastomes carrying mitogenomic DNA.

2. The results are also generally persuasive in demonstrating that NOVOPlasty does not work very well, even for the many "easier" plastomes chosen for this study. Importantly, the authors show that varying Kmer values lead to inconsistent results with NOVOPlasty, which is a major concern. There is one obvious major caveat: It is not clear how well the authors are using NOVOPlasty. For example, all of the Kmer choices seem quite low compared to what I normally use in other software for plastome assembly. In fact, in my experience, higher Kmers inevitably generate more accurate assemblies. Are such low Kmers (23, 31, 39, 47) suggested by NOVOPlasty, or why were they chosen? Were any higher kmers tried?

3. The mitogenome section is much less convincing. Roughly 50% of genomes were completed, but no comparison is made relative to published genomes, so it is unclear whether the GetOrganelle assemblies are reasonable. Also, it is unclear how these results compare to other assembly approaches. Some basic characterization of these genomes is needed, and a discussion of GetOrganelle results relative to other mitogenome assembly approaches is needed.

4. Mitogenomes of four animals and one fungus have very low mapped mito coverage (<20), and unsurprisingly, they did not assemble well. Is this a problem with the GetOrganelle software failing to properly recognize mito reads in these data set? Or is this a fundamental issue with the data sets lacking sufficient mito reads? If this is an issue with the software, more discussion is needed. If the problem lies with the data sets themselves, then they should probably be removed

Some minor comments:
5. p. 3 lines 35-38: The number of plastid genomes (and probably mitogenomes) is very out of date. Moreover, the provided weblink only reports RefSeq genomes, which is just a fraction of the plastomes and mitogenomes actually "available in GenBank". This statement needs to be revised to be more accurate

6. p. 3 lines 45-46: The authors state that WGS assembly "approaches are computationally intensive and rarely assemble complete organelle genomes". This is rather misleading. In reality, it is quite trivial to get complete plastid genomes from such approaches, using a small fraction of data (<5M reads) for the vast majority of plants. The plastid contigs are easy to identify in the assembly, and there are generally just one to a few contigs that that can be easily assembled into a complete plastome. That being said, inexperienced users can certainly assemble plastomes poorly, but I would guess that the error rate of automated software such as NOVOPlasty are more likely to produce incorrect results. The authors need to be more impartial about the different approaches.

7. p. 4 lines 6-7: The authors state that "Freudenthal et al. [24] only tested the plastome assembly with a limited range of applications and parameters using GetOrganelle." However, the same caveat is also probably true for the other software that they analyzed. Rarely do such software comparison manuscripts exhaustively evaluate parameter options, and because of that, most such manuscripts are not very useful. Did Freudenthal et al also use a limited range of parameters for the other evaluated software?

8. p. 8 lines 32-35: The authors state "For SRR2037123, NOVOPlasty produced a problematic assembly with aberrantly high coverage and gain the bestranked matched depths by cheating." This is a poorly worded sentence. NOVOPlasty didn't "cheat". Instead, the method being used to evaluate accuracy, coverage depth, is being led in the specific instance because the assembly is clearly wrong. The authors need to reword this sentence.

**Authors’ response to reviewers**

Reviewer #1:

Dear Colleagues,

I read you manuscript on GetOrganelle with great interest. Although slightly neglected by many genomics labs, the plastome is a crucial element of molecular biology, and should not be discarded with missassembled reads.

You wrote an excellent and convincing paper, which is very clear and organised. It also helps if you can already cite a successful third-party benchmark in your introduction! The discussion is very complete and thoughtful. The code for both the method and the figures are freely available on GitHub.

My only minor comments is that Figure 1, although very creative in its use of graphics, was rather overwhelming to me. In particular, I did not understand how the grey flow fits in the process. Reading through the text, I did not understand why the search for target-like contigs was mapped to green solid arrow 4 and blue solid arrow 2, when these two arrows are different in the figure. Also, the emoticons, although very charming, did confuse me out of context.

*Dear Dr. Zerbino,

Thank you for your positive and encouraging comments.

To answer your question about Figure 1, we wanted to convey that each grey arrow alongside a colored arrow means the corresponding grey step can be independently fulfilled by scripts. We agree with you that blue solid arrow 2 was confusing. In the revised Figure 1, we removed some confusing information to simply the illustration. We also replaced the emoticons with “Assembly succeeds” and “Assembly fails” separately to clarify the meanings.*


I successfully installed the dataset (using pip), but I did not have a test dataset at hand so was not able to test the software completely. Would it be possible for you to include a small test dataset in the installation?

*Our response: Thanks for your suggestion. Now we have provided a test case with a small simulated dataset right after the installation instruction in the GetOrganelle homepage. We added detailed explanations of the running log and results of this test in the GetOrganelle wiki page. Moreover, additional real dataset examples were also provided for the purpose of training potential new users.*


Reviewer #2:

In this study, the authors present a software tool called GetOrganelle that can be used to assemble plastid and mitochondrial genomes. This tool uses five steps, involving the 1) filtration of the read data set to keep reads that match to a seed set, 2) extension to identify additional likely reads, 3) de novo assembly of filtered reads, 4) removal of non-target contigs from the assembly, and 5) resolution of repeats and final assembly. Importantly, the authors evaluated the accuracy of assemblies from GetOrganelle and another popular tool (NOVOPlasty), a step that is very often ignored in published organellar genome studies, leading to an abundance of poor-quality genomes in the sequence databases.

Overall, the results are generally persuasive in demonstrating that GetOrganelle works well for plastid genomes. 44 of 50 plastomes agreed very closely or exactly with published sequences. Importantly, multiple reanalyses with different variations of parameters generally produced identical, or nearly identical, plastomes. Using multiple parameter combinations is a procedure I routinely use to verify my own plastome assemblies. The error and coverage analyses persuasively demonstrate that GetOrganelle's assemblies are probably more accurate than many published sequences, which indicate that many published plastomes likely have small errors, and some have major errors. This is an important observation that I have also routinely noted in my own reanalyses of data sets. There is certainly a need for a reliable automated approach for plastome assembly. The other softwared are very much inadequate, and very problematic for the field because they can produce junk assemblies.

*Our response: Thank you for reviewing our manuscript and your supportive comments and suggestions. Indeed, we found that your comments were really inspiring. We have carefully revised the manuscript where necessary as you and the other reviewer suggested. We are dedicated to make GetOrganelle not only accurate and efficient for beginners, but also reliable and professional for experienced users.*

1. One point of contention is that the authors seemed to have intentionally avoided some of the more difficult plastomes, such as in Ericaceae, Geraniaceae, and Pinus, which as the authors point out have an abundance of smaller repeats. Similarly, I don't think the authors tested any plastomes carrying insertions of mitochondrial DNA, such as carrot, milkweed, and bamboo plastomes. I imagine that these plastomes will be problematic for GetOrganelle (and almost certainly for other automated assemblers), but it would have been informative to see how GetOrganelle performs in these instances. At the very least, the authors should comment on the likelihood of success for plastomes carrying mitogenomic DNA.

*Our response:
1) Of the 50 plastome samples, two Picea samples (ERR268390 and SRR5028199) have “weird” plastome (Figure 1A), which is similar to that of Pinus samples we newly tested herein (Figure 1B,C). The assembly graphs of Picea spp. contained four short “inverted/directed” repeats, which can mediate 15 complete circular sequences. By contrary, the assembly graphs of Pinus spp. contained five short “inverted/directed” repeats, which can export 21 complete circular sequences. Moreover, plastomes of Ericaceae and Geraniaceae have high number of “inverted/directed” repeats as showing in plastome assembly graphs (e.g. Figure 1D), which can produce more than 10 thousand circular sequences. Due to the limitation of short-read sequencing data, it is intractable to identify which sequence is the correct plastome or the dominate type in a plant cell. As the rapid development of sequencing technology, it is hoped that the long-read sequencing data could apply to disentangle the complicate repeat puzzle of the “weird” plastomes in the future assembling methods.*

*2) As the reviewer suggested, we have now conducted ad hoc tests on samples of carrot (Daucus carota, DQ898156 vs. SRR2147183) (Figure 2A), and milkweed (Asclepias_syriaca, KF386166 vs. SRR4243000) (Figure 2B), which generated accurate plastomes with mitochondrial insertions, as we expected from our algorithmic design. Back to the algorithm, in the plastome mode, GetOrganelle keeps both chloroplast contigs and mitochondrial contigs in the post-slimmed assembly graph, so that chloroplast and mitochondrial contigs can be more accurately and reasonably distinguished based on not only blast hit, but also contig coverage and contig connections. If there is strong coverage or graph character evidence for a mitochondrial insertion to be an indispensable part of the plastome, GetOrganelle will keep that insertion. We also added related comments to the manuscript.

In the case of carrot (DQ898156 vs. SRR2147183), the GetOrganelle-assembled SRR2147183 plastome (Figure 2A) differs from the published plastome (DQ898156) in accumulatedly-374-bp small SNPs/indels (Figure 3A). Exactly the same 126-bp mitochondrial insertion mentioned by Goremykin et al (2009) also appeared in the GetOrganelle-assembled plastome in the same location.

In the case of milkweed (KF386166 vs. SRR4243000), the GetOrganelle-assembled SRR4243000 plastome (Figure 2B) differs from the published plastome (KF386166) in accumulatedly-22-bp SNPs/indels, in addition to 102-bp IR boundary difference (Figure 3B). Exactly the same 2427-bp mitochondrial insertion detected by Straub et al (2013) also appeared in the GetOrganelle-assembled plastome in the same location. Besides, the rpl2-like sequence (rpl2 pseudogene) of the insertion was also recorded in the output CSV file along the assembly graph.

We did not find public raw reads for Pariana bamboo species which are reported to have mitochondrial insertion (Ma et al, 2015). Instead, we tested Phyllostachys edulis (HQ337796 vs. SRR8271208) and find GetOrganelle-assembled plastome of SRR8271208 (Figure 2C) to be largely congruent with the published plastome (HQ337796) with only accumulatedly-10-bp small SNPs/indels (Figure 3C). The assembled plastome did not contain detectable mitochondrial insertions, although the assembly graph seemed to be a mess (Figure 2C).*


*Figure 1. Some complicate assembly graphs with several repeats in Pinaceae (A-C) and Ericaceae (D). Contigs are in random colors.


Figure 2. Some post-slimmed assembly graphs of the mixture of chloroplast DNA contigs and mitochondrial DNA contigs in carrot (A), milkweed (B), and bamboo (C). According to the post-assembly comparison with published plastome, GetOrganelle correctly identified the chloroplast DNA contigs (SSC in blue, LSC in red, IRs in green) and mitochondrial DNA contigs (grey). The GetOrganelle-assembled plastomes included exactly the same mitochondrial insertion as the published ones.


Figure 3. Dotplots (word size=15) showing the synteny between the published plastome and GetOrganelle-assembled plastomes.*

2. The results are also generally persuasive in demonstrating that NOVOPlasty does not work very well, even for the many "easier" plastomes chosen for this study. Importantly, the authors show that varying Kmer values lead to inconsistent results with NOVOPlasty, which is a major concern. There is one obvious major caveat: It is not clear how well the authors are using NOVOPlasty. For example, all of the Kmer choices seem quite low compared to what I normally use in other software for plastome assembly. In fact, in my experience, higher Kmers inevitably generate more accurate assemblies. Are such low Kmers (23, 31, 39, 47) suggested by NOVOPlasty, or why were they chosen? Were any higher kmers tried?

*Our response: We believe that we are skillfully using NOVOPlasty to conduct plastome assembly. The NOVOPlasty developers posted that “it's recommended to lower the K-mer (set between 21-39)!!!” at the NOVOPlasty homepage. We tried higher K-mers following your suggestion and found worse results (please find the new results at: https://github.com/Kinggerm/GetOrganelleComparison/tree/master/res/NOVOPlasty-plastome). The NOVOPlasty developers’ strongly recommended K-mer values are most suitable for NOVOPlasty. We followed the NOVOPlasty developers’ official suggestions on other procedures when conducted plastome assembly using NOVOPlasty.*

3. The mitogenome section is much less convincing. Roughly 50% of genomes were completed, but no comparison is made relative to published genomes, so it is unclear whether the GetOrganelle assemblies are reasonable. Also, it is unclear how these results compare to other assembly approaches. Some basic characterization of these genomes is needed, and a discussion of GetOrganelle results relative to other mitogenome assembly approaches is needed.

*Our response: We agreed with you that this part was largely unexplored. We have added the NOVOPlasty assemblies as a comparison to the GetOrganelle assemblies to the revised manuscript now. However, we could not find enough animal and fungal datasets which had both the raw reads and the corresponding assembled mitogenome based on the same individual. As the animal and fungal mitogenomes are fast evolving, we suggest that mitogenomes of the same species, but not the same individual, may not be ideal for comparison. So, we did not include published mitogenomes in this section. Based on the comparison with NOVOPlasty assemblies, GetOrganelle assemblies seemed to be reasonable. Besides, by checking the GetOrganelle produced concomitant cognominal TAB-formatted file recording the contig label table, we also roughly checked whether the commonly appeared 14 or more coding genes showed up in the assembled contigs and found them consistently existed. In addition, we have received positive feedbacks from colleagues and users working on the mitogenome using GetOrganelle.
Although we conducted the comparison with NOVOPlasty assemblies, a more detailed multi-dimensional comparison of mitogenome assemblies, which would need collaborations with colleagues working on animals and fungi to enrich the default Label database, may be more convincing, but is not be the focus of current manuscript. As a precaution, we have added the statement to the revised manuscript that the biological characteristics of GetOrganelle mitogenome assemblies remain to be explored.*

4. Mitogenomes of four animals and one fungus have very low mapped mito coverage (<20), and unsurprisingly, they did not assemble well. Is this a problem with the GetOrganelle software failing to properly recognize mito reads in these data set? Or is this a fundamental issue with the data sets lacking sufficient mito reads? If this is an issue with the software, more discussion is needed. If the problem lies with the data sets themselves, then they should probably be removed

*Our response: When there’s a problem with the GetOrganelle in recognizing mito reads/contigs, it’s most likely the limitation of its default databases. One typical case as described in the manuscript was SRR1822107, where there’s a relative species in Seed database for GetOrganelle to recognize the mito reads, but there’s no that species in the Label database for GetOrganelle to recognize any mito contigs. We have reshaped GetOrganelle in the latest version, where the default database was isolated as an independent repository with version numbers (https://github.com/Kinggerm/GetOrganelleDB), so that the database would be easily evolvable and recordable. Until more comprehensive fungal & animal databases are built up, GetOrganelle will have a small chance to fail in assembling fast evolving animal & fungal mitogenomes, resulting in NO contigs at all (as already warned users in each running log). But if there’s ever contigs been assembled, especially when the accumulated contig length reaches similar to a complete genome, the low coverages issue would more likely be the caused by the data itself. The samples you pointed out are data set issue, which can also be corroborated by the NOVOPlasty assemblies (no contigs in four animals and highly fragmented in one fungus).

We tend not to remove those cases because they are still informative as part of the comparisons and may reflect some cases that most users will meet in real situations.*

Some minor comments:
5. p. 3 lines 35-38: The number of plastid genomes (and probably mitogenomes) is very out of date. Moreover, the provided weblink only reports RefSeq genomes, which is just a fraction of the plastomes and mitogenomes actually "available in GenBank". This statement needs to be revised to be more accurate
 *Our response: Thank you for pointing out this issue. We agreed that the database we cited was not optimal for supporting this statement. Now we downloaded all the associated data from Genbank, removed the duplicates, and revised the numbers and narrative.*

6. p. 3 lines 45-46: The authors state that WGS assembly "approaches are computationally intensive and rarely assemble complete organelle genomes". This is rather misleading. In reality, it is quite trivial to get complete plastid genomes from such approaches, using a small fraction of data (<5M reads) for the vast majority of plants. The plastid contigs are easy to identify in the assembly, and there are generally just one to a few contigs that that can be easily assembled into a complete plastome. That being said, inexperienced users can certainly assemble plastomes poorly, but I would guess that the error rate of automated software such as NOVOPlasty are more likely to produce incorrect results. The authors need to be more impartial about the different approaches.

*Our response: Sorry for the misleading sentence. We have revised the narrative accordingly.*

7. p. 4 lines 6-7: The authors state that "Freudenthal et al. [24] only tested the plastome assembly with a limited range of applications and parameters using GetOrganelle." However, the same caveat is also probably true for the other software that they analyzed. Rarely do such software comparison manuscripts exhaustively evaluate parameter options, and because of that, most such manuscripts are not very useful. Did Freudenthal et al also use a limited range of parameters for the other evaluated software?

*Our response: We are sorry that we had not expressed our meaning correctly. We believe Freudenthal did their analyses professionally, as far as we know. They not only sent emails asking for author’s preference of parameters (our suggestion for GetOrganelle is using every default) but also publically left messages to every software repository. What we wanted to express was that the broad application and versatile options of GetOrganelle and its algorithms were not explored by that benchmark test, which is reasonable for a benchmark test but not for a paper presenting GetOrganelle in detail. We have revised our narrative in the manuscript.*

8. p. 8 lines 32-35: The authors state "For SRR2037123, NOVOPlasty produced a problematic assembly with aberrantly high coverage and gain the bestranked matched depths by cheating." This is a poorly worded sentence. NOVOPlasty didn't "cheat". Instead, the method being used to evaluate accuracy, coverage depth, is being led in the specific instance because the assembly is clearly wrong. The authors need to reword this sentence.

*Our response: We totally agree with you on this and have reworded this sentence now.


Reference

1. Goremykin VV, Salamini F, Velasco R, Viola R. Mitochondrial DNA of Vitis vinifera and the issue of rampant horizontal gene transfer. Mol Biol Evol. 2009;1:99-110.
2. Straub SCK, Cronn RC, Edwards C, Fishbein M, Liston A. Horizontal transfer of DNA from the mitochondrial to the plastid genome and its subsequent evolution in milkweeds (Apocynaceae). Genome Biol Evol. 2013;10:1872–1885.
3. Ma PF, Zhang YX, Guo ZH, Li DZ. Evidence for horizontal transfer of mitochondrial DNA to the plastid genome in a bamboo genus. Sci Rep. 2015;11608.*

**Second round of review**

**Reviewer 2**

The revised manuscript is much improved, and the authors have addressed my previous comments.

I have just two very minor issues:
1) p 11 lines 17-19: The phrase "seems to be a big issue" feels too informal, and perhaps a bit harsh. The authors should more clearly state their concern, and perhaps more diplomatically.
2) p 5, line 9 (and elsewhere): "directed repeats" in the plastome should be called "direct repeats".

I also noticed some typos in the supplementary material. All supplementary files should be checked for more:
3) Table S1, column G header: plastform >> platform
4) Additional File 1, Figure S1 legend: reassemblied >> reassembled

**Authors’ response to reviewers**

We revised the minor issues and corrected the typos following the comments of reviewer #1.
